# SARS-CoV-2 ORF3c impairs mitochondrial respiratory metabolism, oxidative stress, and autophagic flux

**DOI:** 10.1016/j.isci.2023.107118

**Published:** 2023-06-14

**Authors:** Alessandra Mozzi, Monica Oldani, Matilde E. Forcella, Chiara Vantaggiato, Gioia Cappelletti, Chiara Pontremoli, Francesca Valenti, Diego Forni, Marina Saresella, Mara Biasin, Manuela Sironi, Paola Fusi, Rachele Cagliani

**Affiliations:** 1 Scientific Institute IRCCS E. MEDEA, Bioinformatics, 23842 Bosisio Parini, Italy; 2 Department of Biotechnology and Biosciences, University of Milano-Bicocca, 20126 Milano, Italy; 3 Scientific Institute IRCCS E. MEDEA, Laboratory of Molecular Biology, 23842 Bosisio Parini, Italy; 4 Department of Biomedical and Clinical Sciences "L. Sacco", University of Milan, 20157 Milan, Italy; 5 Don C. Gnocchi Foundation ONLUS, IRCCS, Laboratory of Molecular Medicine and Biotechnology, 20148 Milan, Italy

**Keywords:** Virology, Cell biology

## Abstract

Coronaviruses encode a variable number of accessory proteins that are involved in host-virus interaction, suppression of immune responses, or immune evasion. SARS-CoV-2 encodes at least twelve accessory proteins, whose roles during infection have been studied. Nevertheless, the role of the ORF3c accessory protein, an alternative open reading frame of ORF3a, has remained elusive. Herein, we show that the ORF3c protein has a mitochondrial localization and alters mitochondrial metabolism, inducing a shift from glucose to fatty acids oxidation and enhanced oxidative phosphorylation. These effects result in increased ROS production and block of the autophagic flux. In particular, ORF3c affects lysosomal acidification, blocking the normal autophagic degradation process and leading to autolysosome accumulation. We also observed different effect on autophagy for SARS-CoV-2 and batCoV RaTG13 ORF3c proteins; the 36R and 40K sites are necessary and sufficient to determine these effects.

## Highlights

• ORF3c localizes to the mitochondria
• ORF3c acts by modifying mitochondrial metabolism
• ORF3c enhances oxidative stress and mitochondrial ROS production
• ORF3c causes a block of autophagic flux by affecting lysosomal acidification

## Graphical abstract

**Figure:**
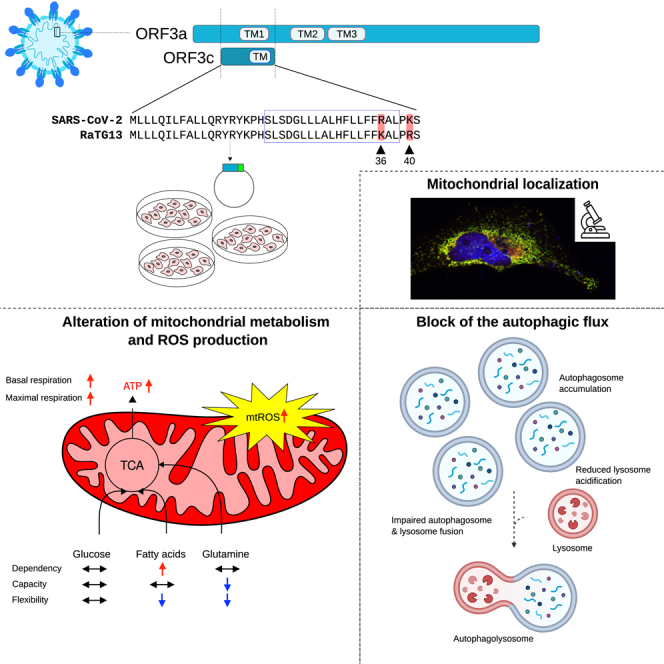

## Introduction

The ongoing COVID-19 pandemic, which is caused by a newly emerged coronavirus (SARS-CoV-2), has to date resulted in more than 6.9 million deaths worldwide (https://covid19.who.int/). Although vaccines have been demonstrated to be highly efficient in preventing severe disease presentation and mortality,^1^ the emergence of new viral variants indicates the need for a deeper understanding of SARS-CoV-2 pathogenic mechanisms, in order to improve prevention and treatment.^2^

SARS-CoV-2 is an enveloped virus consisting of a positive-sense, single-stranded RNA genome of about 30 kb.^3^^,^^4^ Two overlapping ORFs, ORF1a and ORF1b, are translated from the positive-strand genomic RNA and generate continuous polypeptides, which are cleaved into a total of 16 nonstructural proteins (NSPs). The remaining genomic regions encode four structural proteins - spike (S), envelope (E), membrane (M), and nucleocapsid (N) - and six annotated accessory proteins (ORF3a, 6, 7a, 7b, 8, and 10; reference GenBank: NC_045512.2). Also, studies that aimed to evaluate the coding capacity of SARS-CoV-2 identified several unannotated accessory ORFs, including several alternative open reading frames within ORFs S (ORF2d), N (ORF9b, ORF9c), and ORF3a (ORF3b, ORF3c, ORF3d).^5^

Protein-protein interaction data between SARS-CoV-2 proteins and cellular molecules were obtained using different methods, such as affinity purification, proximity labeling-based strategies, and yeast two-hybrid systems.^3^^,^^4^^,^^6^^,^^7^^,^^8^^,^^9^ These host-virus interactome analyses uncovered several human proteins that physically associate with SARS-CoV-2 proteins and that may participate in the virus life cycle, infection, replication, and budding. Among these, interactions with mitochondrial proteins seem to be particularly abundant.^3^^,^^6^^,^^8^ In line with these findings, recent studies suggested the involvement of mitochondria in SARS-CoV-2 infection as a hallmark of disease pathology.^10^^,^^11^^,^^12^^,^^13^ Indeed, recent evidence revealed alterations of mitochondrial dynamics (i.e., increased fusion and inhibition of mitochondrial fission) in patients with COVID-19.^14^ These observations are also consistent with the notion that SARS-CoV-2 infection involves two stages, characterized by different metabolic features.^15^ A first hyper-inflammatory phase, characterized by increased aerobic glycolysis (Warburg effect), mitochondrial dysfunction, and hyperglycemia, is associated with high virus levels and occurs as the host tissues react to the virus by increasing energy production and by activating the innate immune response. This is the phase which often culminates with the cytokine storm.^16^^,^^17^ A second hypo-inflammatory, immune-tolerant phase is associated with a much lower virus level and is characterized by decreased oxygen consumption, resumption of mitochondrial respiration and ATP production, as well as by increased fatty acid oxidation.^18^^,^^19^

In this respect, the study of accessory proteins with mitochondrial localization is of great importance to identify therapeutic targets and to understand the mechanisms of SARS-CoV-2-induced disease.^20^ Indeed, although accessory proteins are considered non-essential for coronavirus replication, accumulating evidence demonstrates that they are critical to virus-host interaction, affecting host innate immunity, autophagy, and apoptosis, as well as contributing significantly to pathogenesis and virulence.^21^ For instance, the ORF9b protein, which localizes to the mitochondria, antagonizes type I and III interferons by targeting multiple innate antiviral signaling pathways.^22^ Another mitochondrial accessory protein, ORF10, inhibits the cell innate immune response by the induction of mitophagy-mediated MAVS degradation.^23^

A notable exception among SARS-CoV-2 accessory proteins is accounted for by ORF3c, which has remained uncharacterized and under-investigated. The ORF3c protein has been predicted to be encoded by sarbecoviruses (a subgenus of betacoronaviruses) only,^24^^,^^25^ including SARS-CoV-2, SARS-CoV, and bat coronavirus RaTG13 (one of the bat betacoronavirus most closely related to SARS-CoV-2^26^). Analysis of the conservation of ORF3c in sarbecoviruses, together with ribosome-profiling data, strongly suggest that ORF3c is a functional protein.^5^^,^^24^^,^^25^^,^^27^ Herein, we report the first investigation of the effect of ORF3c on autophagy and lung cell mitochondrial metabolism.

## Results

### ORF3c protein structure

**Figure 1:**
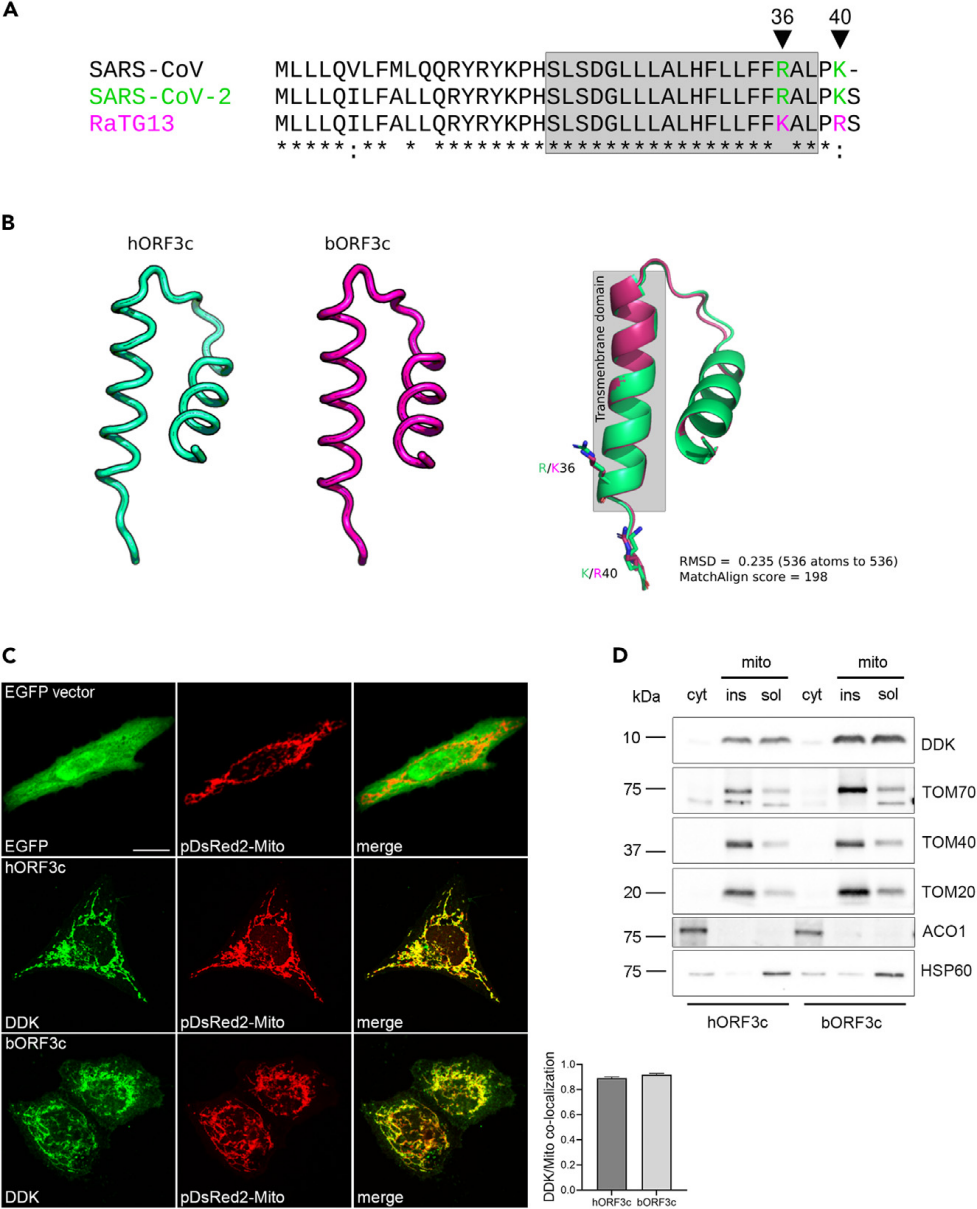
ORF3c localizes to the mitochondria (A) ClustalW alignment of SARS-CoV-2 ORF3c (hORF3c), batCoV RaTG13 ORF3c (bORF3c) and SARS-CoV ORF3c proteins. Transmembrane domains predicted by Phobius (https://phobius.sbc.su.se/) are in gray. The amino acid positions 36 and 40 specific for hORF3c and bORF3c are marked in green and magenta, respectively. (B) Protein structures of hORF3c and bORF3c modeled with the RoseTTAFold software. Superimposition of the two structures is also reported and visualized with PyMOL. (C) Mitochondrial localization of ORF3c proteins. HeLa cells were co-transfected with pDsRed2-Mito vector and pCMV6 hORF3c or bORF3c. Twenty-four hours later, cells were fixed and immunostained with antibodies against the DDK tag. Co-localization (yellow) of ORF3c (green) with mitochondria (red) is shown in the merged images. Pearson’s correlation coefficient (PCC) for the co-localization of DDK and Mito staining is reported in the graph (*n*>20 cells). Scale bar: 10 μm. (D) HeLa cells transiently expressing hORF3c or bORF3c were lysed and total cell extracts were subjected to cellular fractionation. Aliquots of cytosolic and mitochondrial (soluble/insoluble) fractions were analyzed by SDS-PAGE and Western blotting. hORF3c and bORF3c were detected using an anti-DDK antibody. Antibodies directed against the cytosolic protein aconitase 1 (ACO1), the outer mitochondrial membrane translocase subunits TOM20, TOM40 and TOM70, and the mitochondrial matrix heat shock protein 60 (HSP60) were used as markers of the specific cellular compartment/organelle.

SARS-CoV-2 ORF3c (also known as ORF3h) is a 41 amino acid (aa) protein encoded by an alternative open reading frame within the ORF3a gene.^24^^,^^25^^,^^27^ It is highly conserved in sarbecoviruses showing 90% and 95% identity with the corresponding proteins encoded by SARS-CoV and batCoV RaTG13 (Figure 1A). This latter was isolated from horseshoe bats (*Rhinolophus affinis*), a putative reservoir host.^28^

As previously reported, ORF3c has a predicted highly conserved transmembrane domain^27^ (Figure 1A), which suggests interactions within the lipid bilayer.^21^ However, other protein domains have not been described and the protein structure is not available.

We thus modeled the structure of the SARS-CoV-2 and batCoV RaTG13 ORF3c proteins with the RoseTTAFold software using the deep-learning algorithm.^29^ ORF3c structure prediction revealed a tridimensional architecture composed of two short alpha-helices (α1 and α2) connected by a loop region (Figure 1B). The α2 helix corresponds to the predicted transmembrane region. SARS-CoV-2 and RaTG13 ORF3c proteins differ only in two amino acids: R36K (in the predicted transmembrane domain) and K40R (Figure 1A). Structural superposition revealed good conservation of the global protein architecture between the two models (Figure 1B), suggesting that amino acid differences between the two ORF3c proteins do not result in conformational changes.

### ORF3c localizes to the mitochondria

ORF3c subcellular localization was investigated by confocal microscopy. In particular, 123 bp sequences corresponding to the ORF3c of SARS-CoV-2 and RaTG13 (hereafter hORF3c and bORF3c, respectively) were cloned into a mammalian expression vector (pCMV6) in frame with the DDK (FLAG) tag. HeLa cells were transiently transfected with the vectors expressing hORF3c and bORF3c and stained with anti-DDK antibody to detect the viral protein, as well as with antibodies against specific markers of the endoplasmic reticulum, Golgi, lysosomes or early endosomes (Figure S1). For the staining of mitochondria, cells were transfected with the pDsRed2-Mito vector. Immunofluorescence analysis revealed that both hORF3c and bORF3c strongly co-localized with mitochondria (Figure 1C) but not with other cellular markers (Figure S1). A mitochondrial localization was already reported for other SARS-CoV-2 accessory proteins, such as ORF9b.^30^ This latter was previously shown to directly interact with the outer mitochondrial membrane protein TOM70 (translocase of outer membrane 70),^30^ which forms the translocon complex with other TOM proteins.^31^ We found that hORF3c and bORF3c proteins co-localize with TOM70 and TOM20 (Figures S2A and S2B). However, a direct interaction between the two ORF3c proteins and the TOM complex (TOM70, TOM20, and TOM40) was excluded by immunoprecipitation analysis (Figure S2C).

The mitochondrial localization of both ORF3c proteins was confirmed in A549 and HSAEC1 lung cell lines (Figure S3), deriving from lung carcinomatous tissue and normal lung tissue, respectively. Also, we verified that tag (HA or FLAG) does not influence the localization of ORF3c (Figure S4).

Fractionation analysis in HeLa cells confirmed that hORF3c and bORF3c were almost exclusively found in the mitochondria, in both soluble and insoluble (membrane) fractions (Figure 1D). These data indicate that ORF3c localizes in the mitochondria and suggest that, at least partially, the protein product of ORF3c localizes on mitochondrial membranes. Our results are in line with recently published evidence.^32^ Taken together these data suggest that the ORF3c protein targets the mitochondrial outer membrane (MOM) via its predicted transmembrane domain. Such a localization may be promoted by the interaction with PGAM5 and MAVS,^32^^,^^33^ which, in turn, localize to the mitochondrial membrane.

### The SARS-CoV-2 ORF3c protein induces an increase in mitochondrial respiratory metabolism, a reduction in glycolysis and a metabolic shift toward dependency on fatty acids

Because the ORF3c protein localizes to the mitochondria, we investigated whether it acts by modifying mitochondrial metabolism.

The mitochondrial functionality of HSAEC1 cells (healthy lung epithelial cells) transfected with hORF3c, bORF3c, or with the empty vector as a control were investigated through Agilent Seahorse XF Mito Stress analysis (Figure S5A). The use of healthy cells is mandatory in Seahorse analysis; thus, the tumor cell lines HeLa and A549 were excluded from the experiments due to their impaired metabolism.

**Figure 2:**
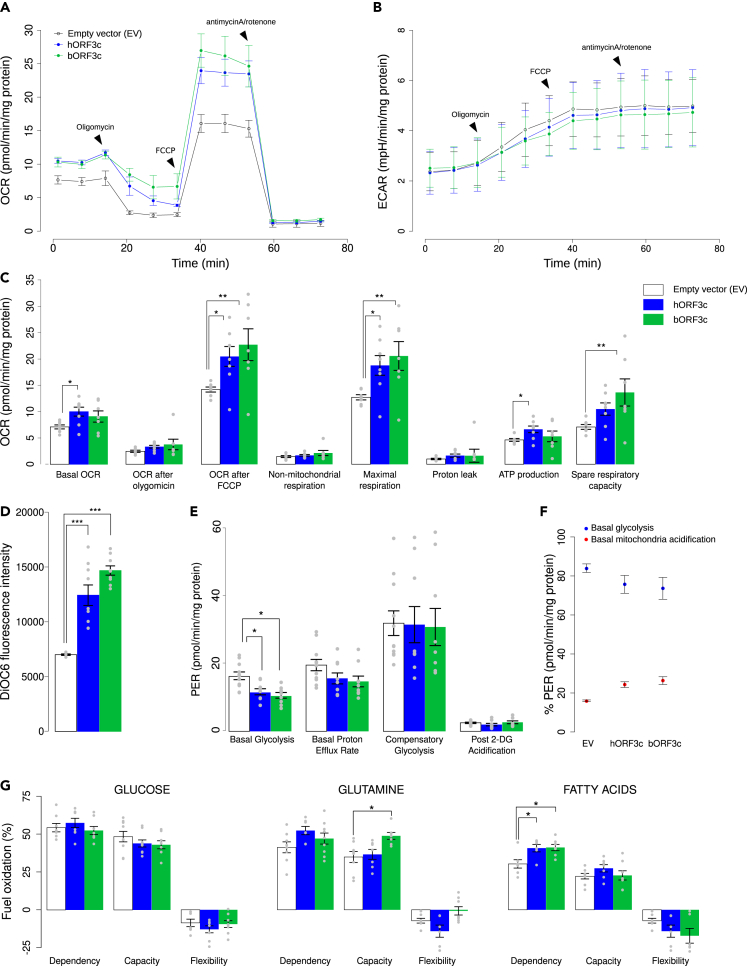
ORF3c modifies mitochondrial metabolism (A) Seahorse mitostress analysis in HSAEC1 cells transfected with hORF3c or bORF3c or the empty vector. Experiments were performed 36h after transfection. OCR traces are expressed as pmoles O_2_/min/mg proteins. Each point was acquired by the Seahorse instrument every 8 min; the arrows indicate the time-points of oligomycin, FCCP and antimycinA/rotenone addition. The OCR profile is representative of four independent experiments, each performed in duplicate. (B) ECAR traces are expressed as mpH/min/mg proteins. The arrows indicate the time-point of oligomycin, FCCP and antimycinA/Rotenone addition. The ECAR profile is representative of three independent experiments, each performed in triplicate. (C) Bars (mean ± SEM) indicate the values at points 3 (basal OCR), 6 (OCR after oligomycin), 9 (OCR after FCCP) and different parameters related with mitochondrial function (non-mitochondrial respiration, maximal respiration, proton leak, ATP production, spare respiratory capacity). Statistical significance was assessed by one way ANOVA followed by Dunnett’s multiple comparison test (n = 8 experiments; ∗p<0.05, ∗∗p<0.01). (D) Analysis of mitochondrial Δψ. After transfection, cells were incubated with 40 nM DiOC6 and the level of fluorescence was evaluated (one way ANOVA followed by Dunnett’s multiple comparison test; n = 9 experiments; ∗∗∗p<0.001). (E) Seahorse glycolytic analysis. Analysis of different parameters related with glycolysis (basal glycolysis, basal proton efflux rate, compensatory glycolysis, post-2DG acidification) (one way ANOVA followed by Dunnett’s multiple comparison test; n = 9 experiments; ∗p<0.05). (F) Proton Efflux Rate (PER) due to glycolysis and to oxidative phosphorylation (one way ANOVA followed by Dunnett’s multiple comparison test; n = 9 experiments). (G) Evaluation of mitochondrial fuel oxidation in HSAEC1 cells transfected with ORF3c from either SARS-CoV-2 or RaTG13, as well as with the empty vector. Glucose, glutamine and long-chain fatty acids mitochondrial fuel oxidation dependency, capacity and flexibility were assayed. Bars indicate the mean ± SEM (one way ANOVA followed by Dunnett’s multiple comparison test; n = 9 experiments; ∗p<0.05). In the plots, only significant comparisons are reported.

The oxygen consumption rate (OCR) and extra-cellular acidification rate (ECAR) profiles are reported in Figures 2A and 2B. In particular, results obtained by measuring real-time OCR showed that the hORF3c protein increases both basal and maximal respiration, as well as mitochondrial ATP synthesis (Figures 2A and 2C). However, this was not matched by an increase in glycolysis, since no differences were observed among ECAR profiles (Figure 2B). An increase in both maximal respiration and spare respiratory capacity was observed in HSAEC1 cells overexpressing the RaTG13 ORF3c protein, whereas the increase in basal respiration was not statistically significant (Figure 2C). Moreover, cells transfected with hORF3c or bORF3c showed a slight increase in oxygen consumption after oligomycin addition (Figure 2C). Although this result may be correlated with mitochondrial uncoupling, the mitochondria of cells overexpressing viral ORF3c proteins are not uncoupled (Figure S5B). Mitochondrial Δψ, measured using a DiOC6 (3,3′-dihexyloxacarbocyanine iodide) fluorescent probe, was found to be more negative in both transfected cells compared to the control (Figure 2D), suggesting oxidative phosphorylation hyperactivation.

In the XF Seahorse Glycolysis Rate Assay, we observed a decrease in the level of basal glycolysis in transfected cells, as well as a decreasing trend in the basal proton efflux rate (PER) (Figure 2E). PER percentage allows us to distinguish between basal mitochondria acidification, due to CO_2_ release, and glycolytic acidification, due to lactic acid production. The overexpression of each ORF led to an increase of the PER derived from mitochondria and a decrease in glycolytic PER (Figure 2F). In accordance, the activity of lactate dehydrogenase (LDH) did not significantly increase after transfection (Figure S5C), suggesting that pyruvate is predominantly used in the Krebs cycle.

We next investigated mitochondria dependence on various substrates through the Seahorse Mito Fuel Flex Test Kit. In particular, cell dependency, capacity, and flexibility in the oxidation of three mitochondrial fuels, namely glucose (pyruvate), glutamine (glutamate), and long-chain fatty acids, were measured using inhibitors of each metabolic pathway (which were injected in a different order and combination). Figure 2G shows the three fundamental parameters for each source of energy. When we analyzed the role of glucose as an energy source, no difference was detected in terms of dependence, capacity, and flexibility between transfected cells and the control. However, when we analyzed glutamine as an energy source, inhibiting the two alternative pathways, cells transfected with bORF3c showed a significant increase in capacity in comparison with both cells transfected with the empty plasmid and cells overexpressing hORF3c. In addition, cells transfected with bORF3c showed an increase in flexibility compared to cells transfected with hORF3c. These cells, therefore, seem to be able to adapt their metabolism by exploiting other fuels when the glutamine pathway is blocked by the BPTES (bis-2-(5-phenylacetamido-1,3,4-thiadiazol-2-yl) ethyl sulfide) inhibitor. On the other hand, cells overexpressing hORF3c protein displayed a slight increase in glutamine dependence compared to the control, and a significant decrease in flexibility compared to bORF3c. This result indicates that the mitochondria of these cells are unable to bypass the blocked pathway by oxidizing other fuels. When fatty acids were investigated as an energy source, cells overexpressing both ORF3c proteins exhibited a significantly higher dependence compared to the control, as shown in Figure 2G. In conclusion, the mitochondria of transfected cells were not only unable to bypass a block of the fatty acid pathway through the use of the other two fuels, but they also required fatty acids to maintain basal OCR.

### Hyperactivation of oxidative phosphorylation is sustained by fatty acid oxidation

**Figure 3:**
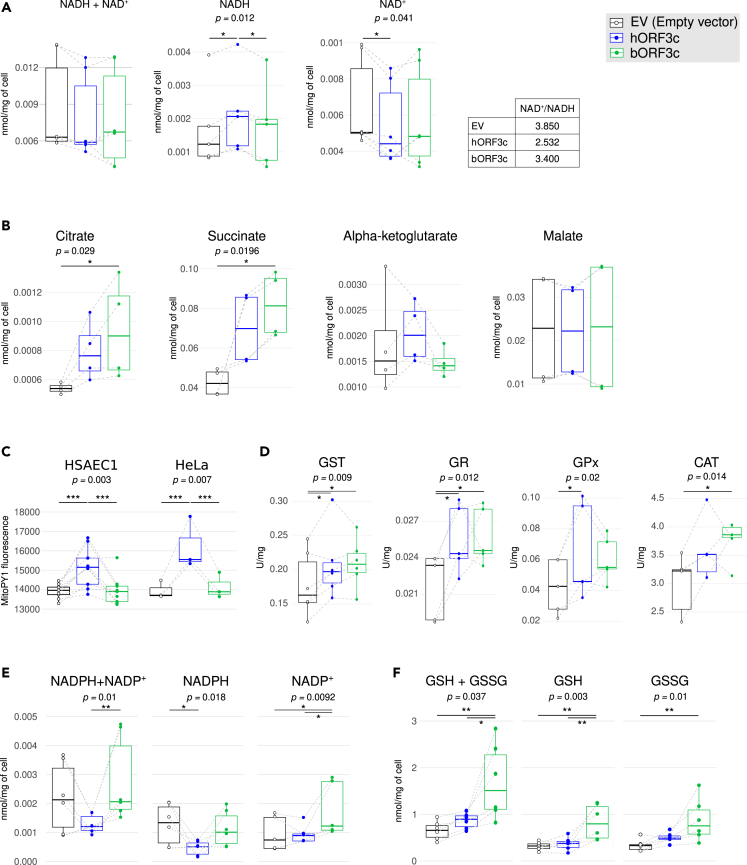
ORF3c induces oxidative stress and increases succinate levels (A) NADH + NAD^+^, NADH and NAD^+^ levels. In the table the relative NAD^+^/NADH ratio is reported, as calculated after NADH and NAD^+^ concentration measurements, in HSAEC1 cells overexpressing either hORF3c or bORF3c proteins, as well as in HSAEC1 cells transfected with the empty vector. Data are presented as boxplot; data referring to the same experiment are linked by a gray dotted line. Statistical significance was assessed by two-way ANOVA followed by Tukey’s multiple comparison test (n = 6 experiments; ∗p<0.05). (B) Analysis of Krebs cycle intermediate levels in HSAEC1 cells transfected with hORF3c or bORF3c, as well as in HSAEC1 cells transfected with an empty vector as a control. Metabolite concentrations were expressed as nmol/mg of cell (two-way ANOVA followed by Tukey’s multiple comparison test; n = 4 experiments; ∗p<0.05). (C) Analysis of mitochondrial H_2_O_2_ production in HSAEC1 and HeLa cells transfected with ORF3c from either SARS-CoV-2 or RaTG13 and in cells transfected with the empty vector. Cells were stained with 5 μM MitoPY1 and the level of cell fluorescence was measured (two-way ANOVA followed by Tukey’s multiple comparison test; HSAEC1: n = 9, HeLa: n = 3; ∗∗∗p<0.001). (D) Activities of enzymes involved in oxidative stress defense. Enzyme activities were measured at saturating substrate concentrations in HSAEC1 cells overexpressing either hORF3c or bORF3c proteins, as well as in HSAEC1 cells transfected with the empty vector (two-way ANOVA followed by Tukey’s multiple comparison test; n ≥ 4; ∗p<0.05). (E) NADPH + NADP^+^, NADPH and NADP^+^ levels in HSAEC1 cells overexpressing either hORF3c or bORF3c proteins, as well as in HSAEC1 cells transfected with the empty vector (two-way ANOVA followed by Tukey’s multiple comparison test; n = 5 experiments; ∗p<0.05, ∗∗p<0.01). (F) Total glutathione (GSH + GSSG), reduced glutathione (GSH) and oxidized glutathione (GSSG) levels measured in HSAEC1 cells overexpressing hORF3c or bORF3c proteins as well as in HSAEC1 cells transfected with the empty vector (two-way ANOVA followed by Tukey’s multiple comparison test; n = 5 experiments; ∗p<0.05, ∗∗p<0.01). All these measures were assayed 36 h after transfection. Only significant comparisons are reported.

Based on Seahorse analysis, we investigated the role of NAD^+^/NADH ratio as the regulator between mitochondrial fatty acid synthesis and oxidation.^34^ In general, fatty acid β-oxidation starts in the presence of an abundant phosphate acceptor and with the consumption of NADH, which leads to an increase in the NAD^+^/NADH ratio. Conversely, during fatty acid synthesis the phosphate acceptor is lacking, while the substrate is present in excess, and most NAD^+^ is reduced. The overexpression of hORF3c protein increased NADH and reduced NAD^+^, leading to a marked decrease in the NAD^+^/NADH ratio (Figure 3A). A smaller, not statistically significant decrease in the ratio was also observed in cells overexpressing bORF3c (Figure 3A). These results indicate that cells transfected with hORF3c increase not merely their use of fatty acids as a carbon source, but also their rate of fatty acid synthesis, to maintain the equilibrium between catabolism and anabolism. A change in NAD^+^/NADH ratio, that is only a mediator of the equilibrium between fatty acid oxidation and synthesis, needs to be supported by the presence of Krebs Cycle substrates. In particular, succinate is the only substrate that can reduce a large pool of mitochondrial NAD^+^ and keep it reduced, whereas citrate could support fatty acid synthesis. Higher levels of citrate and succinate were observed after transfection with either viral proteins (Figure 3B). At the same time, the amount of malate and alfa-ketoglutarate did not reveal any differences between samples.

Because the increase in mitochondrial oxygen consumption due to succinate accumulation can be related to an upregulated mitochondrial subunit content, we used Real-Time PCR to investigate the level of transcripts coding for the various subunits of the five respiratory complexes. We did not detect any significant increase in the level of transcripts in cells transfected with either hORF3c or bORF3c proteins compared to cells carrying the empty plasmid (Figure S5D). COXIII and CytB genes showed a slight increase in expression following transfection with hORF3c (Figure S5D).

The increase in succinate level may be linked to Reverse Electron Transport (RET).^35^^,^^36^ This condition allows cells to use part of the electron flow from succinate to reverse electron transfer through complex I, reducing NAD^+^ to NADH, while another part of the electron flow follows the canonical pathway from CoQ to complex IV and oxygen reduction. The hypothesis seems to be verified only in cells transfected with hORF3c because, as well as a reduction of NAD^+^ to NADH, saturating levels of succinate also lead to a quick conversion of ADP to ATP, and high mitochondria membrane potential, as previously shown. Moreover, the rate of ROS production, especially hydrogen peroxide (H_2_O_2_), in RET is very high.^37^

### ORF3c expression enhances oxidative stress

To further investigate the RET hypothesis, mitochondrial hydrogen peroxide generation was measured using MitoPY1. Results showed that the overexpression of hORF3c, but not of bORF3c, leads to an increase in mitochondrial H_2_O_2_ production in both HeLa and HSAEC1 cell line models (Figure 3C).

In order to evaluate the effect of the overexpression of hORF3c (and bORF3c) proteins in the context of the oxidative stress response induced by an increase of H_2_O_2_, we assayed the activities of different antioxidant enzymes involved in ROS detoxification: glutathione S-transferase (GST) conjugates reduced glutathione with numerous substrates; glutathione reductase (GR) catalyzes the reduction of glutathione disulfide (GSSG) to glutathione (GSH) using NADPH as an electron donor; glutathione peroxidase (GPx) and catalase (CAT) catalyze the decomposition of hydrogen peroxide to water and oxygen. As shown in Figure 3D, the overexpression of hORF3c and bORF3c proteins led to a significant increase in the enzyme activity of GST and GR compared to the control; a significant increase of GPx and CAT were instead observed only in the presence of hORF3c and bORF3c, respectively (Figure 3D).

Although mammalian cells have evolved antioxidant enzymes to protect against oxidative stress, the most important factor in H_2_O_2_ elimination is the availability of NADPH. Indeed, this substrate is required for the regeneration of reduced glutathione, used by GPx and GST, through GR. As reported in Figure 3E a significant decrease of NADPH was observed in the presence of hORF3c with respect to the control. Conversely, bORF3c induced a significant increase in NADP^+^. Glutathione assays showed that the total glutathione level was significantly higher after transfection with bORF3c (Figure 3F).

These data support the idea that cells transfected with the hORF3c protein are not able to adequately eliminate accumulated hydrogen peroxide, whereas cells transfected with bORF3c, although showing some mild signs of oxidative stress, are able to buffer its negative effects thanks to the presence of a sufficient amount of ROS scavengers.

### SARS-CoV-2 ORF3c counteracts autophagy

Mitochondria are most commonly associated with energy production through oxidative phosphorylation, but they are also involved in a myriad of other functions, including innate immune responses.

Upon the infection of a target cell, SARS-CoV-2 may be recognized by innate immunity sensors inducing signaling cascades that lead to the release of IFNs and pro-inflammatory cytokines, as well as to the activation of autophagy for the lysosomal degradation of virus/viral component.^38^^,^^39^

SARS-CoV-2 has evolved a wide variety of strategies to disarm innate host defenses.^39^ For instance, it can alter mitochondrial functions leading to enhanced ROS production, perturbed signaling, and blunted host antiviral defenses. In this respect, an important role is played by accessory proteins, including ORF9b and ORF10, which, such as ORF3c, have a mitochondrial localization.^22^^,^^23^^,^^30^

The function of ORF3c on the antiviral innate immune response was recently reported.^32^^,^^33^ We observed that SARS-CoV-2 ORF3c overexpression induces an increase of ROS. It is known that high levels of mitochondrial ROS can compromise lysosomal acidity and autophagic flux.^40^ Thus, we explored whether ORF3c affects autophagy, an evolutionary conserved intracellular process that delivers proteins and organelles to the lysosomes for degradation, through the formation of double-membrane vesicles, termed autophagosomes. Autophagy is also a key mechanism adopted by the host cell for clearing pathogens. To promote their survival and replication, many viruses, including SARS-CoV-2, have evolved mechanisms to interfere with the formation or maturation of autophagosomes in host cells.^41^^,^^42^

**Figure 4:**
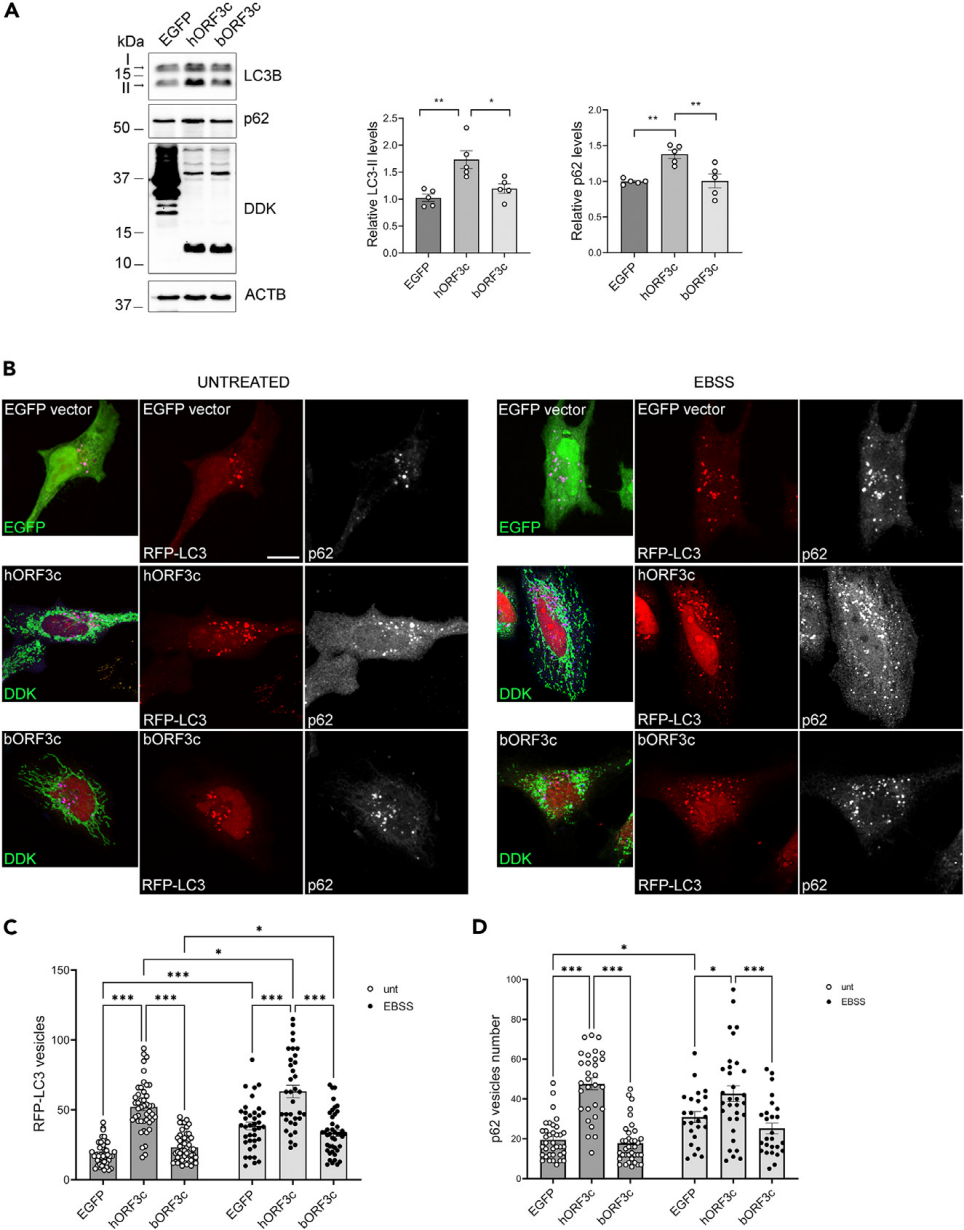
ORF3c overexpression increases autophagosome levels (A) HeLa cells were transfected with hORF3c, bORF3c or a control vector (EGFP). Twenty four hours after transfection cells were lysed and total protein extracts were run onto 10/15% SDS-polyacrylamide gels and probed with anti-DDK, -LC3B, -p62/SQSTM1 and -ACTB Abs. LC3-II and p62 levels were quantified, normalized on ACTB levels and expressed as fold increase of control (one way ANOVA followed by Dunnett’s multiple comparison test; n = 5 experiments; ∗p<0.05, ∗∗p<0.01). (B) Cells were co-transfected with hORF3c, bORF3c or a control vector (EGFP) and the pCMV6-MAP1LC3B-RFP vector for the staining of autophagosomes (red). After 24h, cells were starved in EBSS for 1h to induce autophagy. Treated and untreated cells were fixed and stained with an anti-DDK Ab (green) to detect ORF3c proteins, and with anti-p62 (blue) Abs. Scale bar: 10 μm. (C and D) RFP-LC3 positive vesicles and (D) p62 positive vesicles are reported in the graphs (two-way ANOVA followed by Tukey’s multiple comparison test, n > 25 cells; ∗p<0.05, ∗∗∗p<0.001). Only significant comparisons are reported.

Thus, we analyzed the levels of the autophagosomal markers LC3 and p62 protein, the latter targeting poly-ubiquitinated proteins to autophagosomes for degradation, in ORF3c-transfected cells. During autophagosome formation, the cytosolic LC3-I isoform is converted into an active phosphatidylethanolamine-conjugated form, LC3-II, that is incorporated in the autophagosomal membrane. Thus, LC3-II amount is considered a reliable autophagosomal marker.^43^ Therefore, HeLa cells were transfected with vectors expressing hORF3c, bORF3c, or with the control vector expressing the EGFP-DDK tag, and total protein extracts were analyzed. We found that hORF3c induced an increase in LC3-II and p62 levels (Figure 4A) compared with the control, indicating the presence of an increased number of autophagosomes. Conversely, bORF3c did not affect the levels of autophagosomal markers. Data were confirmed by immunofluorescence by using the pCMV6-MAP1LC3B-RFP vector to stain autophagosomes (Figure 4B). Indeed, we found that, in basal conditions, cells transfected with hORF3c presented autophagosome accumulation with an increased number of RFP-LC3/p62 vesicles (Figures 4C and 4D) compared with control and bORF3c-transfected cells. This effect is independent of the tag used to reveal the viral protein (Figure S4).

Notably, hORF3c also induced autophagosome accumulation in autophagy-inducing conditions. In fact, although starvation with EBSS (Earle’s Balanced Salt Solution) induced autophagy in all transfected cells, the number of autophagosomes remained significantly higher in hORF3c-transfected cells (Figure 4B).

hORF3c and bORF3c only differ by two amino acids, at position 36 and 40 (Figure 1A). To verify the effect of each substitution on autophagy, we mutagenized hORF3c at positions 36 and 40 (R36K and K40R), generating two plasmids: hORF3-36K and hORF3c-40R. We found that the substitutions 36K and 40R individually do not lead to a significant increase in the number of RFP-LC3 vesicles compared to the control (Figure S6A). This suggests that both the 36R and 40K substitutions are necessary and sufficient to determine the accumulation of autophagosomes observed in SARS-CoV-2 ORF3c transfected cells. The effect of hORF3c, bORF3c and of the two substitutions 36K and 40R on autophagosome accumulation were also confirmed in the HSAEC1 cell line (Figure S6B).

**Figure 5:**
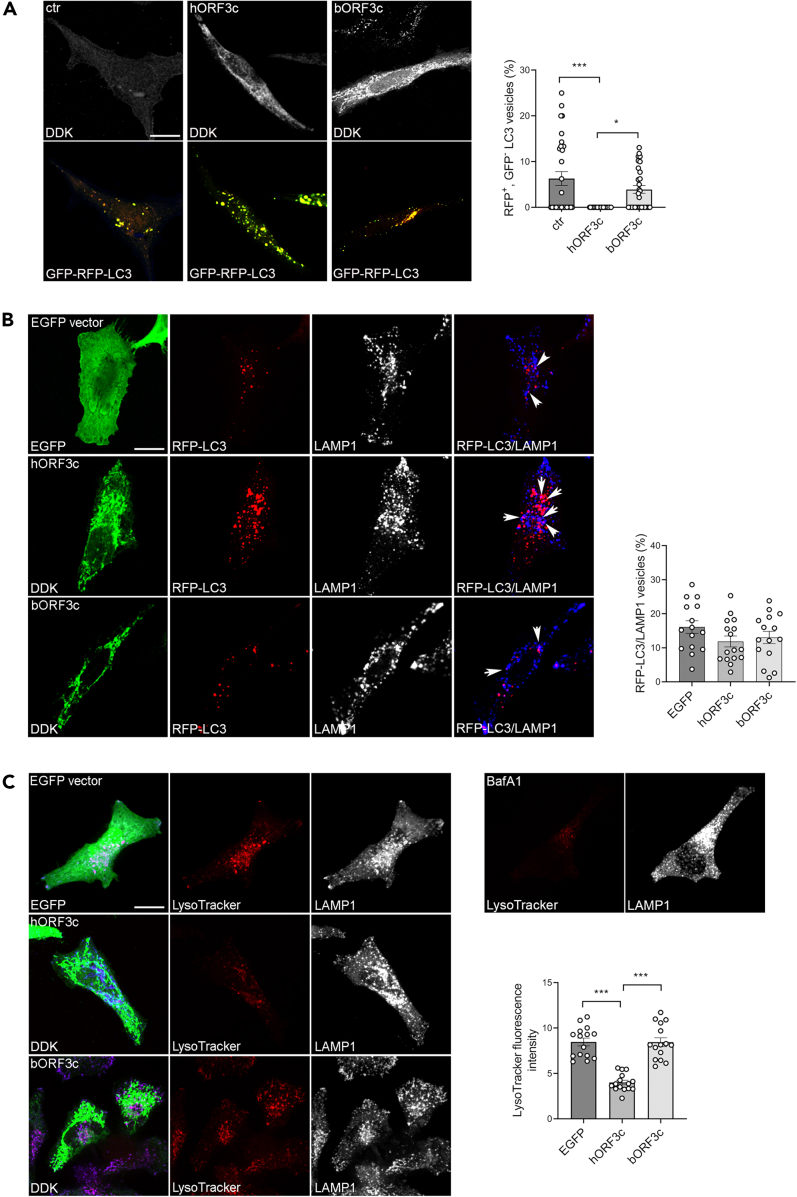
ORF3c overexpression impacts on autophagic flux (A) HeLa cells were co-transfected with mRFP-GFP-LC3 and hORF3c or bORF3c or empty (ctr) vector for 24 h, fixed and stained with an anti-DDK Ab. mRFP-GFP-LC3 positive autophagosomes are shown in yellow. Scale bar, 10 μm. Red mRFP^+^, GFP^−^ LC3 vesicles, corresponding to acidified autolysosomes, were counted and expressed as percentage of total LC3 vesicles (one way ANOVA followed by Dunnett’s multiple comparison test; n = 30 cells; ∗p<0.05, ∗∗∗p<0.001). (B) HeLa cells co-transfected with RFP-LC3B and hORF3c, bORF3c or EGFP vector were stained with Abs against DDK tag (green) and the lysosomal marker LAMP1 (blue). Autophagosomes (RFP-LC3) fused with LAMP1 positive vesicles were counted, normalized to total RFP-LC3 vesicles and expressed as percentage (one way ANOVA followed by Dunnett’s multiple comparison test; n = 15 cells). (C) HeLa cells transfected with hORF3c, bORF3c or EGFP vector were labeled with LysoTracker red DND-99, fixed and immunostained with anti-LAMP1Ab (blue). Scale bar: 10 μm. Bafilomycin A1 (BafA1) was used as negative control. LysoTracker fluorescence intensity was quantified and reported in the graph (one way ANOVA followed by Dunnett’s multiple comparison test; n = 15 cells; ∗∗∗p<0.001). Only significant comparisons are reported.

An increased number of autophagosomes may derive from an increased biogenesis or from the inhibition of the autophagic flux. Therefore, we analyzed autophagosome degradation by using the mRFP-GFP tandem fluorescent tagged LC3B vector to visualize autophagosomes (Figure 5A).^44^ The GFP signal is sensitive to the acidic compartment and is quenched under low-pH conditions when autophagosomes fuse with lysosomes. We found that, compared with cells transfected with the control or with bORF3c, a very low percentage of the autophagosomes accumulated in hORF3c-transfected cells are red acidified functional autolysosomes (mRFP+, GFP-) (Figure 5A). This is indicative of degradation defects, as reported for other SARS-CoV-2 proteins (e.g. ORF7a and ORF3a).^38^ Nevertheless, we found that the percentage of RFP-LC3 vesicles co-localizing with the lysosomal marker LAMP1 was similar in all transfected cells and in untransfected controls, suggesting that the expression of hORF3c did not affect autophagosome-lysosome fusion and that the autophagosome accumulation observed in these cells did not derive from fusion defects (Figure 5B).

We next assessed whether hORF3c affects lysosomal acidification by using the acidic organelle marker LysoTracker red, a cell-permeable weak base dye which selectively accumulates in acidified vesicles, such as lysosomes and autolysosomes.^45^ We observed a decrease in LysoTracker red fluorescence intensity in hORF3c-transfected cells compared with the control, indicating a reduced acidity of lysosomes (Figure 5C). No difference was detected between bORF3c-transfected cells and control.

In summary, these data indicate that SARS-CoV-2 ORF3c (but not bORF3c) impairs autophagy; in particular, ORF3c affects lysosomal acidification, thus blocking the normal autophagic degradation process and leading to autophagosome accumulation.

Autophagy also plays an important role in the maintenance of mitochondrial homeostasis. Indeed, the quality control of mitochondria is achieved by balanced actions among mitochondrial biogenesis, mitochondrial dynamics, and mitophagy, a selective autophagy that removes dysfunctional or exceeding mitochondria.^46^ Viruses often hijack mitophagy to enable immune escape and self-replication.^23^^,^^47^^,^^48^ We therefore analyzed the sequestration of mitochondria in the autophagosomes in ORF3c-transfected cells by quantifying the co-localization of RFP-LC3 and the mitochondrial marker TOM20 (Figure S7). We did not detect differences in the percentage of mitochondria co-localizing with autophagosomes among hORF3c, bORF3c, and the control (Figure S7). These data suggest that the ORF3c protein does not impair mitophagy.

## Discussion

Coronaviruses encode a variable number of accessory proteins, which differ in sequence and number even among closely related viruses. These proteins are usually dispensable for viral replication, but often play a role in host-virus interactions, in the suppression of immune responses, or in immune evasion. For these reasons, some of them represent virulence factors.^49^^,^^50^^,^^51^ Therefore, gaining full insight into the functions of accessory proteins is pivotal for understanding coronavirus pathogenesis and for the development of effective antiviral drugs.

Since the beginning of the pandemic, the accessory proteins encoded by SARS-CoV-2 have been an object of study and their role in immune evasion, as well as their interaction with host proteins, have been reported. Although highly conserved in sarbecoviruses and considered a potentially functional protein,^5^^,^^24^^,^^25^^,^^27^ the accessory protein ORF3c of SARS-CoV-2, an alternative open reading frame within the ORF3a gene, attracted little attention. To cover this gap, we characterized ORF3c in terms of cellular localization, autophagy modulation, and effects on mitochondrial metabolism. Our data show that ORF3c has a mitochondrial localization, alters mitochondrial metabolism, and increases ROS production. ORF3c also acts on autophagy by blocking the autophagic flux and inducing the accumulation of autophagosomes/autolysosomes. Recently, two preprints that demonstrate a role for ORF3c in host’s antiviral response modulation were posted.^32^^,^^33^ In particular, these studies show that, through its interaction with MAVS and PGAM5, ORF3c prevents the activation of IFN-beta transcription. Both PGAM5 and MAVS have a role in antiviral signaling and localize to the mitochondrial membrane.^52^^,^^53^

Because the mitochondrial localization of ORF3c may lead to an alteration of mitochondrial functionality, we investigated oxidative metabolism through Seahorse assays. Notably, in pulmonary cell lines overexpressing ORF3c, we observed a decrease in the level of basal glycolysis, paralleled by an increase in maximal respiration and spare respiratory capacity. Thus, we suggest that ORF3c acts by mimicking a condition of glucose starvation, leading to an increased dependency on fatty acids as a fuel. Alterations of cellular metabolism have also recently been reported in cells expressing ORF7a or ORF7b, indicating that accessory proteins may play an important role in these processes.^54^

The metabolic rearrangement induced by ORF3c is reminiscent of events that occur during the second phase of SARS-CoV-2 infection. In the first phase of infection, characterized by high virus levels, the energy supply occurs mainly through the hyperactivation of glycolysis, which culminates with the reduction of pyruvate into lactate. On the other hand, mitochondrial oxidative phosphorylation is very marginal to energy production: the respiratory complexes allow electron transfer with poor efficiency, and the electrochemical potential across the inner mitochondrial membrane is low. This first phase is functional for the replication of the virus and its expansion in the host. The second phase, associated with much lower virus levels, is a chronic degeneration of cellular physiology^15^; at this point, in line with what we observed when transfecting cells with ORF3c, oxidative phosphorylation is the main way of energy production, glycolysis being downregulated. Fatty acids become the primary energy substrate, beta-oxidation being upregulated; glucose consumption and lactate production decrease, reducing acidification. Acetyl-CoA is channeled into the citrate cycle, which proceeds predominantly in the canonical direction. Finally, a shift from glucose oxidation to fatty acid oxidation occurs. Clearly, these changes most likely result from the concerted action of multiple viral proteins. Our data suggest that ORF3c contributes to induce a metabolic shift toward fatty acids oxidation in the presence of glucose. How ORF3c achieves this result remains unclear and further studies are required to establish the mechanism by which the viral protein alters mitochondrial metabolism. Likewise, it is unclear how ORF3c can alter the metabolic state of infected cells. Given its mitochondrial localization, we hypothesize that the ORF3c protein does not act directly on the glycolytic process, but rather on the transport of pyruvate from the cytoplasm to the mitochondrial matrix or in the early stages of pyruvate modification.

The activation of oxidative phosphorylation (OXPHOS) and β-oxidation of fatty acids is known to induce oxidative stress.^36^^,^^55^^,^^56^ In fact, we observed a significant increase of mitochondrial hydrogen peroxide (H_2_O_2_, a non-radical ROS). An increase in ROS has been described in several physiological and pathological conditions including aging, cancer, diabetes, neurodegenerative disorders, and infection.^57^ In most of these cases, high levels of mitochondrial ROS compromise lysosomal acidity and autophagic flux. Recently, it was demonstrated that an increase in ROS levels in glucose-deprived fibroblasts can reduce lysosome acidification and impair autolysosome degradation, eventually blocking the autophagic flux.^40^ Indeed, increased ROS levels might inactivate the vacuolar ATPase (vATPase), a proton pump that generates an acidic pH in the lysosome.^40^

In our study, we observe a block of the autophagic flux in cells ectopically expressing ORF3c. In particular, our data show that ORF3c expression may prevent autophagic degradation by altering lysosomal pH. Altogether, these observations suggest that the alteration of mitochondrial metabolism we observed in ORF3c-transfected cells may be responsible for lysosome deacidification and autophagosome/autolysosome accumulation, as already reported in glucose-deprived fibroblasts.^40^

Interestingly, ORF3c does not affect mitophagy despite its mitochondrial localization. A prevention of mitophagy activation was also shown by Stewart and colleagues.^32^ In their article, the authors reported that ORF3c interacts with PGAM5, a mitochondrial protein that plays a role in upregulating IFN-β signaling during infection^58^ and is involved in mitophagy.^53^ It is possible that ORF3c sequesters PGAM5, thus explaining the observed absence of mitophagy activation.

Autophagic responses can be induced or manipulated by several RNA viruses, which exploit autophagosomes to facilitate viral replication and to elude innate immune responses.^59^ Among these, SARS-CoV-2 restricts autophagy-associated signaling and blocks autophagic flux. In particular, cells infected with SARS-CoV-2 show an accumulation of key metabolites, the activation of autophagy inhibitors, and a reduction in the levels of several proteins responsible for processes spanning from autophagosome formation to autophagosome-lysosome fusion and lysosome deacidification.^60^^,^^61^ Recently, different studies analyzed the effect of individual SARS-CoV-2 proteins on autophagy and identified several viral proteins involved in this process. Some of them act by causing an increase or inhibition in autophagy, but most of the viral proteins (e.g. E, M, ORF3a, and ORF7a) promote the accumulation of autophagosomes, also reducing autophagic flux.^38^^,^^62^ Specifically, ORF3a and ORF7a were reported to block autophagy by interfering with autophagosome-lysosome fusion and lysosomal acidification.^38^^,^^63^^,^^64^^,^^65^^,^^66^ In particular, ORF3a was found to block autophagosome maturation by targeting multiple protein complexes required for autophagosome-lysosome fusion, such as HOPS-mediated SNARE complex and UVRAG-containing PI3KC3 complexes.^63^^,^^64^ Indeed, autophagy inhibition was demonstrated to be extremely critical for the life cycle of SARS-CoV-2 and other human coronaviruses.^67^ Taking all these data together, we suggest that, during SARS-CoV-2 infection, various mechanisms are put in place to regulate autophagy, with the aim to achieve a state of equilibrium that both allows the inhibition of the innate immune response and favors viral replication. In this scenario, it is not surprising that multiple viral proteins can modulate autophagic flux by exploiting different mechanisms in order to remodel the autophagic process to facilitate viral replication.

In this context, an important role is likely to be played by ORF3c, not only in SARS-CoV-2, but probably in all sarbecoviruses, where ORF3c is highly conserved. To test this hypothesis, we evaluated the effect on autophagy of the ORF3c protein encoded by one of the bat betacoronaviruses most closely related to SARS-CoV-2 (batCoV RaTG13, bORF3c). In most analyses, a similar trend as that observed for SARS-CoV-2 ORF3c was evident for bORF3c, but the effect was definitely weaker. The two viral proteins (hORF3c and bORF3c) differ only in two amino acids at position 36 and 40. Our data indicate that the 36R and 40K sites are necessary and sufficient to determine the accumulation of autophagosomes and to justify a different effect on autophagy for SARS-CoV-2 and RaTG13 ORF3c proteins (in our experimental conditions). It is thus tempting to speculate that substitutions in the ORF3c protein also have important effects in the circulating variants of the virus and in particular in some variants of concern (VOC). Interestingly, the Beta variant carries a non-synonymous mutation at position 36 of ORF3c (R36I, corresponding to mutation Q57H in ORF3a). The R36I mutation is predicted to determine a conformational change in the protein structure, without however having any effect on cellular localization and on IFN-suppressive activity.^33^ On the basis of our data it is possible to hypothesize that R36I has instead a specific action on the modulation of autophagy. Specific experiments to evaluate this possibility are thus warranted.

In analogy to other accessory proteins, ORF3c is dispensable for viral replication. In fact, the absence of the protein caused by premature stop codons in different lineages and sublineages (e.g. Q5∗ in delta variant) does not alter viral replication efficiency. Nevertheless, this ORF is highly conserved among sarbecoviruses, suggesting that its physiological role is important for the virus. An interesting possibility is that ORF3c, as well as other accessory proteins, is particularly relevant for infection and virus maintenance in the natural reservoir (i.e., bats).

In summary, ORF3c acts on two fundamental processes: innate immune response and autophagy. Both are dysregulated during SARS-CoV-2 infection and represent the targets of different viral proteins, especially accessory proteins. In this study, we focused on the action of ORF3c on the block of the autophagic flux, showing how overexpression of SARS-CoV-2 ORF3c leads to an accumulation of autophagosomes by reducing lysosome acidification. We also demonstrated that the ORF3c protein determines a modulation of mitochondrial metabolism. To our knowledge, this is the first study in which the effect of a single SARS-CoV-2 protein on mitochondrial metabolism has been evaluated together with its direct effect on the autophagic process. Future studies evaluating the role of SARS-CoV-2 viral proteins (in particular of accessory proteins) that interact directly or indirectly with mitochondria will provide a detailed picture of how SARS-CoV-2 targets this organelle to counteract autophagy and to antagonize type I IFN induction.

### Limitations of the study

The major limitation of this study is the use of an *in vitro* cellular model. In fact, the data obtained (cellular localization, alteration of mitochondrial metabolism, and blockage of autophagic flux) are the results of ectopic expression of the ORF3c protein in commercial cell lines. Conversely, we did not evaluate the localization and cellular functions of ORF3c in the context of SARS-CoV-2 infection.

Moreover, we noted a different action of hORF3c and bORF3c on the block of autophagic flux. We verified that this difference depends on the amino acid composition of the ORF3c proteins encoded by SARS-CoV-2 and RaTG13. We cannot however exclude that the different effect observed for bORF3c is at least partially explained by the use of human cell lines. Thus, another limitation of this study lies in not having tested the effect of bORF3c overexpression in bat cell lines.

## STAR★Methods

### Key resources table

**Table:** 

REAGENT or RESOURCE	SOURCE	IDENTIFIER
**Antibodies**		

Mouse monoclonal anti-DDK – Clone 4C5	OriGene	Cat# TA50011-100, RRID:AB_2622345
Rabbit polyclonal anti-DDK antibody	OriGene	Cat# TA100023 RRID:AB_2622243
Mouse monoclonal anti-HA tag antibody (F-7)	Santa Cruz Biotechnology	Cat# sc-7392 RRID:AB_627809
Rabbit anti-LC3B antibody	Cell Signaling Technology	Cat# 2775, RRID:AB_915950
Rabbit anti-p62 / SQSTM1 antibody	Sigma-Aldrich	Cat# P0067, RRID:AB_1841064
Mouse monoclonal anti-BNIP3 antibody [ANa40]	Abcam	Cat# ab10433, RRID:AB_2066656
Mouse anti-β-Actin Antibody (C4)	Santa Cruz Biotechnology	Cat# sc-47778, RRID:AB_626632
Rabbit polyclonal anti-Aconitase 1 antibody	Proteintech	Cat# 12406-1-AP, RRID:AB_10642942
Rabbit polyclonal anti-TOM20 antibody	Proteintech	Cat# 11802-1-AP, RRID:AB_2207530
Rabbit polyclonal anti-TOM40 antibody	Proteintech	Cat# 18409-1-AP, RRID:AB_2303725
Rabbit polyclonal anti-TOM70 antibody	Proteintech	Cat# 14528-1-AP, RRID:AB_2303727
Mouse monoclonal anti-HSP60 antibody (2E1/53)	Thermo Fisher Scientific	Cat# MA3-013, RRID:AB_325461
Rabbit polyclonal anti-LAMP1	Abcam	Cat# ab24170, RRID:AB_775978
Goat polyclonal anti-EEA1 (N-19)	Santa Cruz Biotechnology	Cat# sc-6415, RRID:AB_2096822
Rabbit polyclonal anti-GM130 (C-terminal)	Sigma-Aldrich	Cat# G7295, RRID:AB_532244
Rabbit polyclonal anti-calreticulin	Thermo Fisher Scientific	Cat# PA3-900, RRID:AB_325990
Goat anti-Mouse IgG (H+L) Cross-Adsorbed Secondary Antibody, Alexa Fluor 488	Thermo Fisher Scientific	Cat# A-11001, RRID:AB_2534069
Goat anti-Rabbit IgG (H+L) Cross-Adsorbed Secondary Antibody, Alexa Fluor 546	Thermo Fisher Scientific	Cat# A-11010, RRID:AB_2534077
Donkey anti-Mouse IgG (H+L) Highly Cross-Adsorbed Secondary Antibody, Alexa Fluor 488	Thermo Fisher Scientific	Cat# A-21202, RRID:AB_141607
Donkey anti-Rabbit IgG (H+L) Highly Cross-Adsorbed Secondary Antibody, Alexa Fluor 546	Thermo Fisher Scientific	Cat# A10040, RRID:AB_2534016
Donkey anti-Goat IgG (H+L) Cross-Adsorbed Secondary Antibody, Alexa Fluor 647	Thermo Fisher Scientific	Cat# A-21447, RRID:AB_2535864
Peroxidase-AffiniPure Goat Anti-Rabbit IgG (H+L)	Jackson ImmunoResearch	Cat# 111-035-003 RRID:AB_2313567
Peroxidase-AffiniPure Goat Anti-Mouse IgG (H+L)	Jackson ImmunoResearch	Cat# 115-035-003 RRID:AB_10015289

**Chemicals, peptides, and recombinant proteins**		

Dulbecco’s Modified Eagle’s Medium (DMEM)	Euroclone	Cat# 41965-039
Fetal Bovine Serum (FBS)	Euroclone	Cat# ECS5000DH
L-glutamine	Invitrogen	Cat# ECB3000D
Penicillin/Streptomycin	Invitrogen	Cat# ECB3001D
SABM Basal Medium	Lonza	Cat# CC-3119
SAGM™ SingleQuots™	Lonza	Cat# CC-4124
Earle’s Balanced Salt Solution (EBSS)	Euroclone	Cat# ECB4055L
Trypsin-EDTA 1X	Euroclone	Cat# ECB3052D
Phosphate-buffered saline (PBS)	Euroclone	Cat# ECB4053L
Lipofectamine 2000 Transfection reagent	Thermo Fisher Scientific	Cat# 11668027
Lipofectamine 3000 Transfection reagent	Thermo Fisher Scientific	Cat# L3000015
Poly-L-lysine hydrobromide	Sigma-Aldrich	Cat# P2636
4% paraformaldehyde	Santa Cruz Biotechnology	Cat# sc-281692
Saponin	Merck Life Science	Cat# S4521
Triton X-100	Merck Life Science	Cat# T8787
Bovine serum albumin (BSA)	Merck Life Science	Cat# A9647
DAPI	Roche	Cat# 10236276001
LysoTracker Red DND-99	Invitrogen	Cat# L7528
CHAPS	Merck	Cat# 26680
Halt™ Protease Inhibitor Cocktail EDTA-free	Thermo Fisher Scientific	Cat# 78425
MitoPY1	Tocris Bioscience	Cat# 4428
DiOC6	Merck	Cat# 318426
SuperScript® II RT	Invitrogen	Cat# 18064-014
SYBR Green PCR Master Mix	Applied Biosystems	Cat# 4309155
Leupeptin	Merck	Cat# L2884
Aprotinin	Merck	Cat# A1153
Pepstatin	Merck	Cat# P5318
NP40	Merck	Cat# 492016
NADH	Merck	Cat# N4505
Piruvate	Merck	Cat# 107360
1-Chloro-2,4-dinitrobenzene	Merck	Cat# 138630
GSH	Merck	Cat# G4251
NADPH	Roche	Cat# 10107824001
GSSG	Merck	Cat# 49740
EDTA	Merck	Cat# E1644
NaN_3_	Merck	Cat# S2002
Glutathione Reductase	Merck	Cat# G3664

**Critical commercial assays**		

Mitochondria Isolation Kit for Cultured Cells	Thermo Fisher Scientific	Cat# 89874
Pierce™ MS-Compatible Magnetic IP Kit, protein A/G	Thermo Fisher Scientific	Cat# 90409
Pierce™ BCA Protein Assay Kit	Thermo Fisher Scientific	Cat# 23225
*In vitro* toxicology assay kit, MTT-based	Merck	Cat# TOX-1KT
Cell Mito Stress Test Kit for Agilent Seahorse XF96	Agilent Technologies	Cat# 103015-100
Glycolytic Rate Assay Kit For Agilent Seahorse XF96	Agilent Technologies	Cat#103344-100
Citrate Assay Kit	Merck	Cat# MAK057
Succinate Colorimetric Assay Kit	Merck	Cat# MAK184
α-ketoglutarate Assay Kit	Merck	Cat# MAK054
Malate Assay Kit	Merck	Cat# MAK067
NAD/NADH Quantitation kit	Merck	Cat# MAK037
NADP/NADPH Quantitation kit	Merck	Cat# MAK038
Glutathione Colorimetric Detection Kit	Invitrogen	Cat# EIAGSHC
RNeasy Mini Kits	Qiagen	Cat# 74104

**Experimental models: Cell lines**		

Human epithelial adenocarcinoma HeLa cells	ATCC	CCL-2
Normal human lung HSAEC1-KT cells	ATCC	CRL-4050
Human epithelial lung carcinoma A549	ATCC	CCL-185

**Oligonucleotides**		

Q-PCR: ND2 Fw, CCAGCACCACAACCCTACTA ND2 Rv, GGCTATGATGGTGGGGATGA	This paper	N/A
cyt b Fw: TGAAACTTCGGCTCACTCCT Rv: CCGATGTGTAGGAAGAGGCA	This paper	N/A
COX I Fw: GAGCCTCCGTAGACCTAACC Rv: TGAGGTTGCGGTCTGTTAGT	This paper	N/A
COX II Fw: ACCGTCTGAACTATCCTGCC Rv: AGATTAGTCCGCCGTAGTCG	This paper	N/A
COX III Fw: ACCCACCAATCACATGCCTA Rv: GTGTTACATCGCGCCATCAT	This paper	N/A
ATP6 Fw: GCCACCTACTCATGCACCTA Rv: CGTGCAGGTAGAGGCTTACT	This paper	N/A
ATP8 Fw: TGCCCCAACTAAATACTACCGT Rv: GGGGCAATGAATGAAGCGAA	This paper	N/A
β-actin Fw: CGACAGGATGCAGAAGGAG Rv: ACATCTGCTGGAAGGTGGA	This paper	N/A
ORF3c-36K Fw:CTTGCTGTTTTTCAAAGCGCTTCCAAAATCA Rv: TGATTTTGGAAGCGCTTTGAAAAACAGCAAG	This paper	N/A
ORF3c-40R Fw: CAGAGCGCTTCCAAGATCAACGCGTACGCGG Rv: CCGCGTACGCGTTGATCTTGGAAGCGCTCTG	This paper	N/A

**Recombinant DNA**		

pCMV6-Entry Mammalian Expression Vector (empty vector)	Origene	Cat# PS100001
pCMV6-hORF3c	Origene	N/A, this paper
pCMV6-bORF3c	Origene	N/A, this paper
pCMV6-EGFP	Origene	N/A, this paper
pCMV6-hORF3c-36K	This paper	N/A
pCMV6-hORF3c-40R	This paper	N/A
pCMV-HA-C	Clontech Laboratories	Cat# 635690
pCMV-HA-C-hORF3c	This paper	N/A
pDsRed2-Mito	Clontech Laboratories	Cat# PT3633-5
pCMV6-RFP-MAP1LC3B	Origene	Cat# RC100053
ptfLC3 vector	Kimura et al, 2007^44^	Addgene plasmid #21074

**Software and algorithms**		

Phobius	Käll et al., 2004^68^	https://phobius.sbc.su.se/
Robetta	Baek et al., 2021^29^	https://robetta.bakerlab.org/
PyMOL,Version 1.8.4.0.	Schrödinger, LLC	https://pymol.org/2/
Fiji ImageJ software	Schneider et al., 2012^69^	https://imagej.nih.gov/ij/
Prism 9.3.0	GraphPad Software	https://www.graphpad.com/scientific-software/prism/

### Resource availability

#### Lead contact

Further information and requests for resources and reagents should be directed to and will be fulfilled by the lead contact, Rachele Cagliani (rachele.cagliani@lanostrafamiglia.it).

#### Materials availability

All unique material generated in this study are listed in the key resources table and available from the lead contact.

### Method details

#### Protein structure prediction

The three-dimensional structures of SARS-CoV-2 and RaTG13 ORF3c proteins were predicted using the Robetta online protein structure prediction server (https://robetta.bakerlab.org/).^29^ Robetta can predict the three-dimensional protein structure given an amino acid sequence. The default parameters were used to produce models using the simultaneous processing of sequence, distance, and coordinate information by the three-track architecture implemented in the RoseTTAfold method.^29^ For both proteins, the confidence of the model was good (*Global Distance Test*, *GTD*, > 0.5). 3D structures were rendered using PyMOL (The PyMOL Molecular Graphics System, Version 1.8.4.0; Schrödinger, LLC). The predicted structural model 1 of the top five models of both proteins were used to perform the structural superposition, using the align command. The RMSD value was also calculated with PyMOL.

#### Plasmids

Complementary DNA (cDNA) containing the coding sequences of ORF3c encoded by SARS-CoV-2 (hORF3c, GenBank: NC_045512.2, nucleotide position: 25457-25579) and RaTG13 (bORF3c, GenBank: MN996532, nucleotide position: 25442-25564) were synthesized by the Origene custom service. hORF3c and bORF3c were cloned in the pCMV6-Entry Mammalian Expression Vector (Origene, PS100001) in frame with C-terminus Myc-DDK tag. Likewise, EGFP was cloned in pCMV6-Entry (pCMV6-EGFP, EGFP vector). hORF3c was also cloned in pCMV-HA-C (Clontech Laboratories, Inc., CA, USA). pCMV6-EGFP and pCMV6-Entry Mammalian Expression Vector (empty vector) were used as controls.

pCMV6-hORF3c-36K and pCMV6-hORF3c-40K constructs were generated by site-direct mutagenesis using Pfu DNA Polymerase (PromegaMadison, WI, USA) and pCMV6-hORF3c as a template. Following site-directed mutagenesis PCR, the template chain was digested using DpnI restriction endonuclease and PCR products were directly used to transform TOP10 *E*. *coli* competent cells (Invitrogen, Carlsbad, CA, USA). Mutagenesis was confirmed through Sanger sequencing.

The commercial expression vectors pDsRed2-Mito (Clontech Laboratories, Inc., CA, USA), pCMV6-RFP-MAP1LC3B (Origene, RC100053) were used for fluorescent labeling of mitochondria and autophagosomes, respectively. To analyse autophagosome degradation, cells were transfected with the mRFP-GFP-LC3 (ptfLC3) vector, a gift from Tamotsu Yoshimori (Addgene plasmid #21074).^44^

#### Cell lines and culture conditions

Human epithelial adenocarcinoma HeLa (ATCC, CCL-2) cells and human epithelial lung carcinoma A549 (ATCC, CCL-185) cells were cultured in Dulbecco’s Modified Eagle’s Medium (DMEM, Euroclone, Milano, Italy) supplemented with 10% Fetal Bovine Serum (FBS, Euroclone, Milano, Italy), 2 mM L-glutamine and 100 U/ml penicillin/streptomycin (Invitrogen, Carlsbad, CA, USA, Thermo Fisher Scientific, Waltham, MA, USA). The normal human lung cell line HSAEC1-KT (ATCC® CRL-4050™) was grown in SABM Basal Medium™ supplemented with Bovine Pituitary Extract (BPE), Hydrocortisone, human Epidermal Growth Factor (hEGF), Epinephrine, Transferrin, Insulin, Retinoic Acid, Triiodothyronine, Bovine Serum Albumin – Fatty Acid Free (BSA-FAF), 100 U/ml penicillin and 100 μg/ml streptomycin. All the reagents for HSAEC1 cell culture were supplied by Lonza (Lonza Group, Basel, Switzerland). Cell lines were maintained at 37°C in a humidified 5% CO_2_ incubator. All cell lines were tested for mycoplasma contamination (MP0035; Merck Life Science).

Autophagy was induced by amino acid and serum starvation in Earle’s Balanced Salt Solution (EBSS, ECB4055L, Euroclone) for the indicated times.

#### Immunostaining and confocal immunofluorescence

HeLa/A549/HSAEC1 cells were seeded (0.3 x 10^5^ cells/well) 24 h before transfection into 6-well plates onto coverslips treated with 0.1 ug/mL poly-L-lysine. Transient transfections were performed using Lipofectamine 2000 (Thermo Fisher Scientific, Waltham, MA, USA) with 2.5 μg of plasmid DNA (pCMV6-hORF3c, pCMV6-bORF3c, pCMV6-Entry, pCMV6-EGFP), according to manufacturer’s instruction. For the staining of autophagosomes and mitochondria, cells were co-transfected with the pCMV6-RFP-MAP1LC3B vector and with the pDsRed2-Mito vector, respectively. Co-transfections were performed with 2 μg of each plasmid. At 24 hours after transfection, cells were fixed with 4% paraformaldehyde (Santa Cruz Biotechnology, sc-281692) and permeabilized with phosphate-buffered saline (PBS; Euroclone, ECB4053L) containing 0.1% saponin (Merck Life Science, S4521) and 1% BSA (Merck Life Science, A9647).Samples were then incubated for 2 h with primary antibodies and revealed using the secondary antibodies Alexa Fluor 488, 546 and 647 (Invitrogen, Thermo Fisher Scientific). Nuclei were stained with DAPI. To analyse autophagosome degradation, cells were transfected with the mRFP-GFP-LC3 (ptfLC3) vector, fixed with cold methanol for 5 min and permeabilized with PBS containing 0.1% Triton X-100 (Merck Life Science, T8787). For the staining of acidic organelles, cells were incubated with 75 nM LysoTracker Red DND-99 (L7528, Invitrogen, Thermo Fisher Scientific) for 5 minutes to avoid alkalinization, accordingly with manufacturer instructions, fixed in paraformaldehyde and processed.

Confocal microscopy was performed with a Yokogawa CSU-X1 spinning disk confocal on a Nikon Ti-E inverted microscope equipped with a Nikon 60x/1.40 oil Plan Apochromat objective and were acquired with an Andor Technology iXon3 DU-897-BV EMCCD camera (Nikon Instruments S.p.A., Firenze, Italy). RFP-LC3, p62 and LAMP1 positive vesicles were counted with ImageJ/Fiji by using the “analyze particles” tool and the investigator was blinded as to the nature of the sample analyzed. Pearson’s correlation coefficients for protein co-localization were determined with ImageJ/Fiji software using the COLOC2 plugin.

#### Mitochondria isolation and fractionation

HeLa cells were seeded (1.2 x 10^6^ cells/well) into p100 plates 24 h before transfection. Transient transfections were performed using Lipofectamine™ 3000 Transfection Reagent (Thermo Fisher Scientific, Waltham, MA, USA) with 15 μg of plasmid DNA/plate (pCMV6-hORF3c and pCMV6-bORF3c), according to the manufacturer’s instruction. 24 h post transfection cells were rinsed twice with PBS and harvested by centrifugation. Mitochondria isolation was performed using the Mitochondria Isolation Kit for Cultured Cells (Thermo Fisher Scientific, Waltham, MA, USA) using the reagent-based method starting from about 2 x 10^7^ cells for each construct, according to the manufacturer’s protocol. For each sample, total extracts were fractionated, separating intact mitochondria from cytosol. After isolation, mitochondria were lysed with 2% CHAPS in 25mM Tris, 0.15M NaCl, pH 7.2 and centrifuged at high speed to separate the soluble fraction (supernatant) to the insoluble fraction (pellet).

#### Co-immunoprecipitation assays

Co-immunoprecipitation assays were performed with the Pierce™ MS-Compatible Magnetic IP Kit, protein A/G (Thermo Fisher Scientific, Waltham, MA, USA). Briefly, 24 h post transfection HeLa cells were rinsed twice with ice-cold PBS and lysed on ice in IP-MS Cell Lysis Buffer added of Halt™ Protease Inhibitor Cocktail EDTA-free (Thermo Fisher Scientific, Waltham, MA, USA), for 10 minutes with periodic mixing. Extracts were clarified by centrifugation (13,000 × g for 10 minutes) and quantified by Pierce™ BCA Protein Assay Kit (Thermo Fisher Scientific, Waltham, MA, USA). 500 μg of cell lysate were combined with 5μg of IP antibody and incubated overnight at 4°C with mixing to form the immune complex. The immunoprecipitation reaction was performed for 1h at RT, by incubating the sample/antibody mixture with 0.25 mg of pre-washed Pierce Protein A/G Magnetic Beads. After washes, target antigen samples were eluted in IP-MS Elution Buffer and dried in a speed vacuum concentrator. Samples were reconstituted in Sample Buffer for SDS-PAGE/WB analyses.

#### SDS-PAGE and western blotting

After 24h post transfection, cells were rinsed with ice-cold PBS, harvested by scraping and lysed in Lysis buffer (125 mM Tris/HCl pH 6.8, 2.5% SDS). Lysates were incubated for 2 min at 95°C. Homogenates were obtained by passing 5 times through a blunt 20-gauge needle fitted to a syringe and then centrifuged at 12,000xg for 8 min. Supernatants were analyzed for protein content by Pierce™ BCA Protein Assay Kit (Thermo Fisher Scientific, Waltham, MA, USA). SDS-PAGE and Western-blot were carried out by standard procedures: samples were loaded and separated on a 10%, 12% or 15% acrylamide/bis-acrylamide gel, blotted onto a nitrocellulose membrane (Amersham, Cytiva, Marlborough, MA, USA). Horseradish peroxidase-conjugated secondary antibodies were used and signals were detected using ECL (GE Healthcare) and acquired with iBrightFL1000 (Thermo Fisher Scientific). Protein levels were quantified by densitometry of immunoblots using ImageJ/Fiji software.

#### Viability assay

In order to evaluate the effect of ORF3c from SARS-CoV-2 or from batCov RaTG13 on cell viability, HSAEC1 cells were seeded in 96-well plates at a density of 1 × 10^4^ cells/well and after 24 h were transiently transfected using Lipofectamine 2000 (Thermo Fisher Scientific, Waltham, MA, USA). After an incubation at 37°C for 36 h post transient transfection, the medium was replaced with complete medium without phenol red and 10 μL of 5 mg/mL MTT solution (*In vitro* toxicology assay kit, MTT-based, TOX-1KT, Merck, Darmstadt, Germany) were added to each well. After a further 4 h incubation time, absorbance upon solubilization was measured at 570 nm using a micro plate reader. Viabilities were expressed as a percentage of the mock (pCMV6-vector). No effect on cell viability was detected.

#### Oxygen consumption rate and extra-cellular acidification rate measurements

Oxygen consumption rate (OCR) and extra-cellular acidification rate (ECAR) were investigated using Agilent Seahorse XFe96 Analyzer on HSAEC1 cell line transfected with ORF3c from SARS-CoV-2 or ORF3c from batCov RaTG13. HSAEC1 cells transfected with the empty vector were used as a control.

Cells were seeded in Agilent Seahorse 96-well XF cell culture microplates at a density of 4 × 10^4^ cells per well in 180 μL of growth medium and after 24 h were transiently transfected.

Before running the assay, the Seahorse XF Sensor Cartridge was hydrated and calibrated with 200 μL of Seahorse XF Calibrant Solution in a non-CO_2_ 37 °C incubator to remove CO_2_ from the media that would otherwise interfere with pH-sensitive measurements.

After 36 h incubation at 37°C post transient transfection, the growth medium was replaced with 180 μL/well of Seahorse XF RPMI Medium, pH 7.4 with 1 mM Hepes, without phenol red, containing 1 mM pyruvate, 2 mM L-glutamine and 10 mM glucose. Subsequently, the plate was incubated into a 37°C non-CO_2_ incubator for 1 hour, before starting the experimental procedure, and the compounds were loaded into injector ports of the sensor cartridge.

For Agilent Seahorse XF Cell Mito Stress Test Kit, pre-warmed oligomycin, FCCP, rotenone and antimycin A compounds were loaded into injector ports A, B and C of sensor cartridge at a final working concentration of 1 μM, 2 μM and 0.5 μM, respectively. OCR and ECAR were detected under basal conditions followed by the sequential addition of the compounds and non-mitochondrial respiration, maximal respiration, proton leak, ATP respiration, respiratory capacity and coupling efficiency were evaluated.

For Agilent Seahorse XF Glycolytic Rate Assay Kit, pre-warmed combination of rotenone and antimycin A at working concentration of 0.5 μM and 2-deoxy-D-glucose (2-DG) at 50 mM were loaded into injector ports A and B, respectively. OCR and ECAR were detected under basal conditions followed by the sequential addition of the compounds to measure basal glycolysis, basal proton efflux rate, compensatory glycolysis and post 2-DG acidification.

Using the Agilent Seahorse XF Mito Fuel Flex Test Kit, the mitochondrial fuel consumption in living cells was determined and, through OCR measuring, the dependency, capacity and flexibility of cells to oxidize glucose, glutamine and long-chain fatty acids was calculated. Pre-warmed working concentration of 3 μM BPTES, 2 μM UK5099 or 4 μM etomoxir were loaded into injector port A and compounds mixture of 2 μM UK5099 and 4 μM etomoxir, 3 μM BPTES and 4 μM etomoxir or 3 μM BPTES and 2 μM UK5099 into injector port B to determine glutamine, glucose and long-chain fatty acid dependency, respectively. On the contrary, fuel capacity was measured by the addition into injector port A of 2 μM UK5099 and 4 μM etomoxir, 3 μM BPTES and 4 μM etomoxir or 3 μM BPTES and 2 μM UK5099 working concentration, followed by injection in port B of 3 μM BPTES, 2 μM UK5099 or 4 μM etomoxir working concentration for glutamine, glucose and long-chain fatty acid, respectively. Data were normalized on total protein content as determined by the Bradford method using BSA for the calibration curve.^70^ All kits and reagents were purchased from Agilent Technologies (Santa Clara, CA, USA).

#### Enzymatic activities and metabolite assays

After 36 h post transfection, HSAEC1 cells overexpressing either human or bat ORF3c protein or transfected with the empty vector (control cells), were rinsed with ice-cold PBS, harvested by scraping and lysed in 50 mM Tris-HCl, pH 7.4, 150 mM NaCl, 5 mM EDTA, 10 % glycerol, 1 % NP40 buffer, containing 1 μM leupeptin, 2 μg/mL aprotinin, 1 μg/mL pepstatin and 1 mM phenylmethylsulfonyl fluoride (PMSF). After lysis on ice, homogenates were obtained by passing the cells 5 times through a blunt 20-gauge needle fitted to a syringe and then centrifuging at 15,000g for 30 min at 4°C. Enzyme activities were assayed on supernatants. Lactate dehydrogenase (LDH) was evaluated measuring the disappearance of NADH at 340 nm according to Bergmeyer.^71^ The protein samples were incubated with 85 mM potassium phosphate buffer, 0.2 mM NADH, 0.6 mM pyruvate. Glutathione S-transferase (GST) was measured as reported in Habig,^72^ using 1 mM reduced glutathione (GSH) and 1 mM 1-chloro-2,4-dinitrobenzene (CDNB) as substrates in the presence of 90 mM potassium phosphate buffer pH 6.5. The reaction was monitored at 340 nm. Glutathione reductase (GR) was measured following the disappearance of NADPH at 340 nm according to Wang.^73^ The protein samples were incubated with 100 mM potassium phosphate buffer pH 7.6, 0.16 mM NADPH, 1 mM EDTA, 1 mg/mL BSA, 4.6 mM oxidized glutathione (GSSG). The glutathione peroxidase (GPx) activity was based on the oxidation of GSH using H_2_O_2_ as substrate, coupled to the disappearance of NADPH by glutathione reductase (GR), according to Nakamura.^74^ The protein samples were incubated with 50 mM sodium phosphate buffer pH 7.5, 0.16 mM NADPH, 1 mM NaN3, 0.4 mM EDTA, 1 mM GSH, 0.2 mM H2O2, 2 U/mL GR. Catalase (CAT) activity was evaluated according to Bergmeyer,^75^ using 12 mM H2O2 as substrate in the presence of 50 mM sodium phosphate buffer, pH 7.5. The reaction was monitored at 240 nm.

Enzyme activities were expressed in international units and referred to protein concentration as determined by the Bradford method using BSA for the calibration curve.^70^

L-citrate, L-succinate, α-ketoglutarate, L-malate, NAD^+^/NADH, NADP^+^/NADPH were evaluated using kits based on colorimetric assays (Citrate Assay Kit, MAK057; Succinate Colorimetric Assay Kit, MAK184; α-ketoglutarate Assay Kit, MAK054; Malate Assay Kit, MAK067; NAD/NADH Quantitation kit, MAK037; NADP/NADPH Quantitation kit, MAK038; Merck, Darmstadt, Germany).

For glutathione detection, cells were trypsinized and harvested by centrifugation at room temperature, for 10 min at 1,200×g. Pellets were washed in 3 mL PBS, harvested by a centrifugation and weighed to normalize the results to mg of cells. Pellets were resuspended in 500 μL cold 5% 5-sulfosalicylic acid (SSA), lysed by vortexing and by passing through a blunt 20-gauge needle fitted to a syringe 5 times. All the samples were incubated for 10 min at 4 °C and then centrifuged at 14,000×g for 10 min at 4°C. The supernatant was prepared and used for the analysis following the instructions of Glutathione Colorimetric Detection Kit (catalog number EIAGSHC, Invitrogen, Carlsbad, CA, USA). The Kit is designed to measure oxidized glutathione (GSSG), total glutathione (GSH + GSSG) and reduced glutathione (GSH) concentrations through enzymatic recycling assay based on glutathione reductase and reduction of Ellman reagent (5,5-dithiobis(2-nitrobenzoic acid)) and using 2-vinylpyridine as reagent for the derivatization of glutathione.^76^ Therefore, it was possible to obtain GSH/GSSG ratio, a critical indicator of cell health. The absorbance was measured at 405 nm using a micro plate reader. The values of absorbance were compared to standard curves (GSH tot and GSSG, respectively) and normalized to mg of cells. Final concentrations were expressed in nmol/mg cells.

#### Detection of mitochondrial hydrogen peroxide

MitoPY1 (Tocris Bioscience, Bristol, UK) indicator was used to detect the mitochondrial hydrogen peroxide production in intact adherent cells. The oxidation of this probe forms intermediate probe-derived radicals that are successively oxidized to generate the corresponding fluorescent products.^77^ HSAEC1 and HeLa cells were seeded in 96-well plates at a density of 1 × 10^4^ cells/well and after 24 h were transiently transfected. After an incubation at 37°C for 36 h post transient transfection, the cells were stained with MitoPY1 at 5 μΜ final concentration in 1 PBS for 20 min in the dark at 37°C. After staining, the cells were washed by warm PBS and the fluorescence (excitation = 485 nm; emission = 528 nm) was measured using a fluorescence microtiter plate reader (VICTOR X3) and analyzed by the PerkinElmer 2030 Manager software for Windows.

#### Mitochondrial transmembrane potential (MTP) assay

MTP alterations were assayed through fluorescence analysis, using the green fluorescent membrane dye 3,3′-dihexyloxacarbocyanine Iodide (DiOC6), which accumulates in mitochondria due to their negative membrane potential and can be applied to monitor the mitochondrial membrane potential. After 36 h post transfection, cells were incubated with 40 nM DiOC6 diluted in PBS for 20 min at 37°C in the dark and rinsed with PBS; after adding PBS, fluorescence was measured (excitation = 484 nm; emission = 501 nm) using VICTOR Multilabel plate reader (PerkinElmer, Waltham, MA, USA).

#### RNA isolation and Q-PCR

Total RNA was isolated from cells using RNeasy Mini Kits (Qiagen, Chatsworth, CA, USA), according to the manufacturer’s instructions. RNA was reverse-transcribed using SuperScript® II RT (Invitrogen, Carlsbad, CA, USA), oligo dT and random primers, according to the manufacturer’s protocol.

For quantitative real-time PCR (Q-PCR), the SYBR Green method was used. Briefly, 50 ng cDNA was amplified with SYBR Green PCR Master Mix (Applied Biosystems, Foster City, CA, USA) and specific primers (100 nM), using an initial denaturation step at 95°C for 10 min, followed by 40 cycles of 95°C for 15 sec and 59°C annealing for 1 min. Each sample was analyzed for NADH dehydrogenase subunit 2 (ND2), cytochrome b (cyt b), cytochrome c oxidase subunit I (COX I), cytochrome c oxidase subunit II (COX II), cytochrome c oxidase subunit III (COX III), ATP synthase F0 subunit 6 (ATP6) and ATP synthase F0 subunit 8 (ATP8) expression and normalized for total RNA content using β-actin gene as an internal reference control. The relative expression level was calculated with the Livak method (2[-ΔΔCt]) and was expressed as fold change ± standard deviation. The accuracy was monitored by the analysis of melting curves.

#### Statistics

Student’s t test for unpaired variables (two-tailed) and one way ANOVA or two-way ANOVA followed by Dunnett’s or Tukey’s multiple comparisons tests were performed using GraphPad Prism version 9.3.0 for Windows, GraphPad Software, San Diego, California USA. In one-way ANOVA, the treatment (transfected plasmid) was entered as the independent variable. For two-way ANOVA, the second independent variable was the experiment (to account for the variability among experimental replicates).

Results are reported as individual data plus the mean ± SEM; *n* represents individual data, as indicated in each figure legend. *p* values of less than 0.05 were considered significant. Individual *p* values are indicated in the graphs (∗*p*<0.05; ∗∗*p*<0.01; ∗∗∗*p*<0.001). The statistical analysis applied in each experiment is reported in the corresponding figure legend.

## Data Availability

Any additional information required to reanalyze the data reported in this paper is available from the lead contact upon request (Rachele Cagliani; rachele.cagliani@lanostrafamiglia.it).
