# Supplementary material for: SARS-CoV-2 ORF3c impairs mitochondrial respiratory metabolism, oxidative stress, and autophagic flux
Source: iScience. 2023 Jun 14;26(7):107118. doi: 10.1016/j.isci.2023.107118 (PMC10265927; doi:10.1016/j.isci.2023.107118)
Supplement: Document S1. Figures S1–S7 [file mmc1.pdf]

iScience, Volume *26*

Supplemental information

SARS-CoV-2 ORF3c impairs mitochondrial respiratory metabolism, oxidative stress, and autophagic flux

Alessandra Mozzi, Monica Oldani, Matilde E. Forcella, Chiara Vantaggiato, Gioia Cappelletti, Chiara Pontremoli, Francesca Valenti, Diego Forni, Marina Saresella, Mara Biasin, Manuela Sironi, Paola Fusi, and Rachele Cagliani

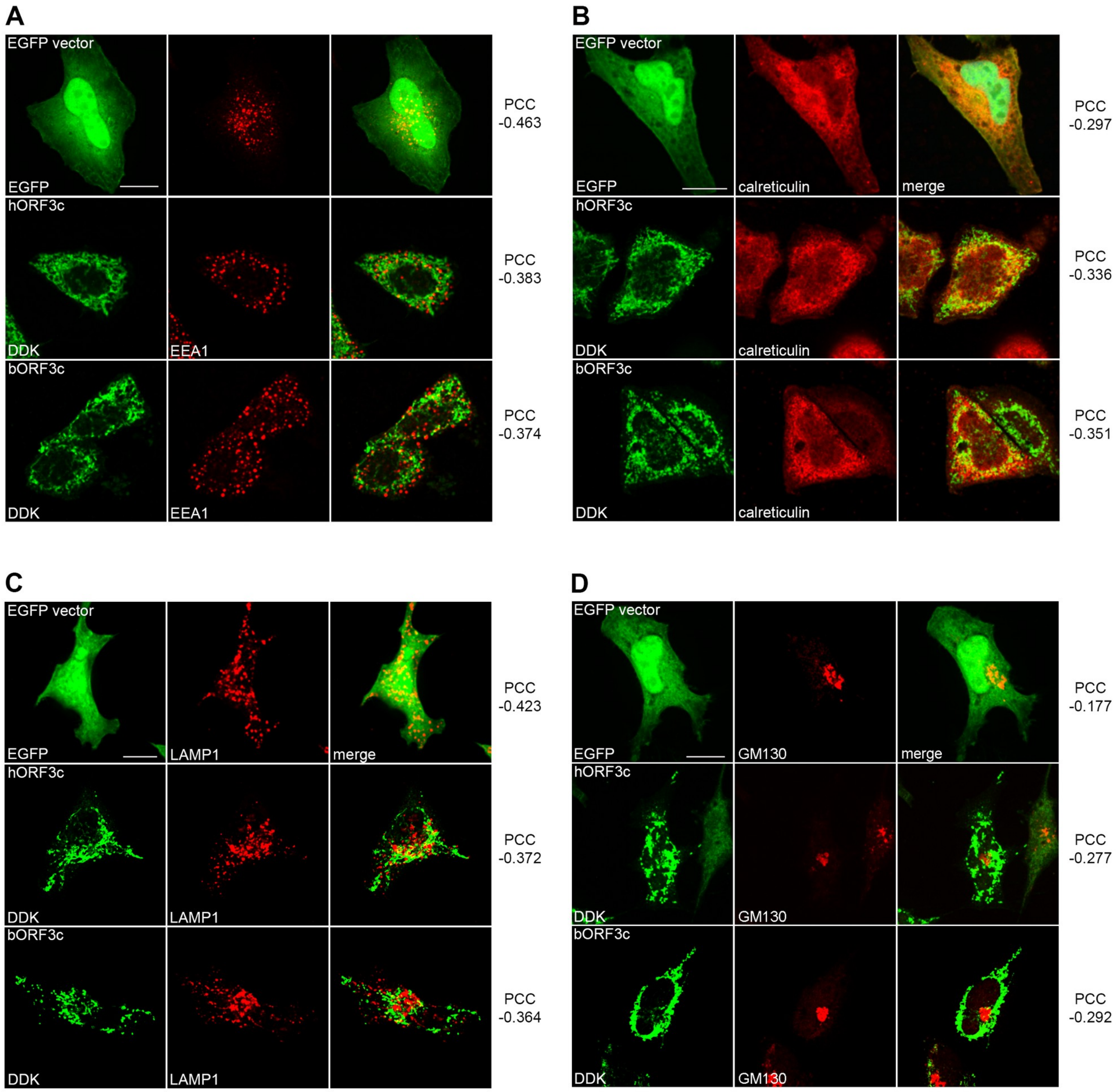

Figure S1. Analysis of ORF3c localization, related to Figure 1.
HeLa cells were transfected with hORF3c, bORF3c or the EGFP control vector. After 24 h, they were stained with antibodies against the DDK tag (green) and (**A**) the early endosomal marker EEA1, (**B**) the endoplasmic reticulum marker calreticulin, (**C**) the lysosomal marker LAMP1 or (**D**) the Golgi marker GM130. Pearson's correlation coefficient (PCC) was negative for all the markers analyzed, indicating no co-localization. Mean Pearson's correlation coefficient from *n*=20 cells are indicated near the respective transfected vector.

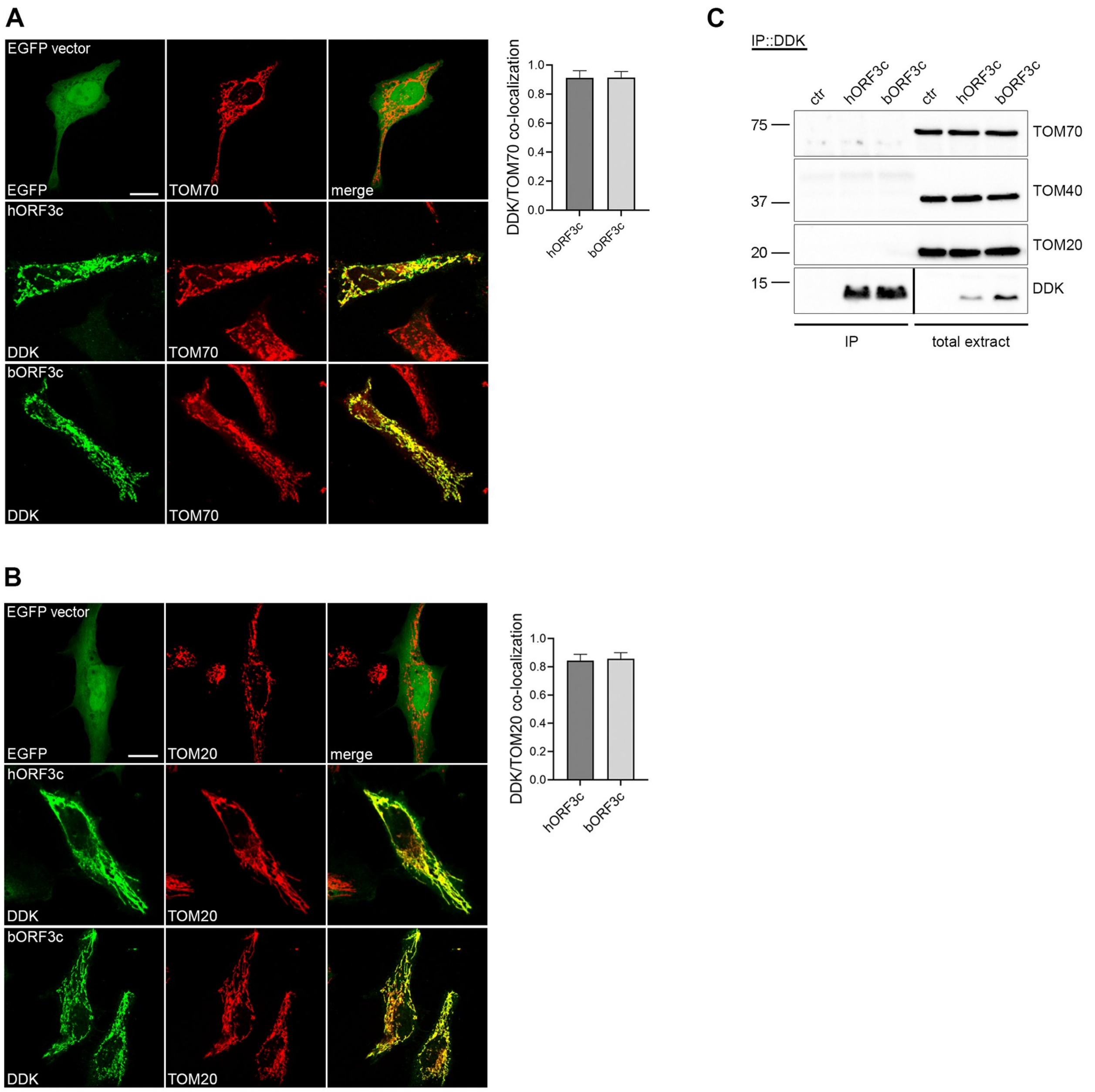

Figure S2. ORF3c proteins co-localize with mitochondrial TOM complex components, related to Figure 1. HeLa cells were transfected with hORF3c, bORF3c or the EGFP control vector. After 24 h, they were stained with antibodies against the DDK tag (green) and TOM70 (red) or TOM20 (red). Co-localization (yellow) of DDK with (**A**) TOM70 or (**B**) TOM20 is shown in the merge images. Scale bar: 10 µm. Pearson's correlation coefficients for DDK/TOM70 and DDK/TOM20 co-localization are reported in the graphs for hORF3c and bORF3c (*n*=20 cells). A negative Pearson's correlation coefficient was obtained for EGFP/TOM proteins co-localization (DDK/TOM70 = - 0.51; DDK/TOM20 = - 0.58). (**C**) Co-immunoprecipitation of endogenous TOM70, TOM20 and TOM40. HeLa cells were transfected with DDK-tagged hORF3c, bORF3 or empty vector (ctr) and after 24 h total protein extracts were subjected to immunoprecipitation (IP) with anti DDK Ab. A representative blot out of three reproducible ones is shown. The black line indicates lanes that were run on the same gel but were non-contiguous.

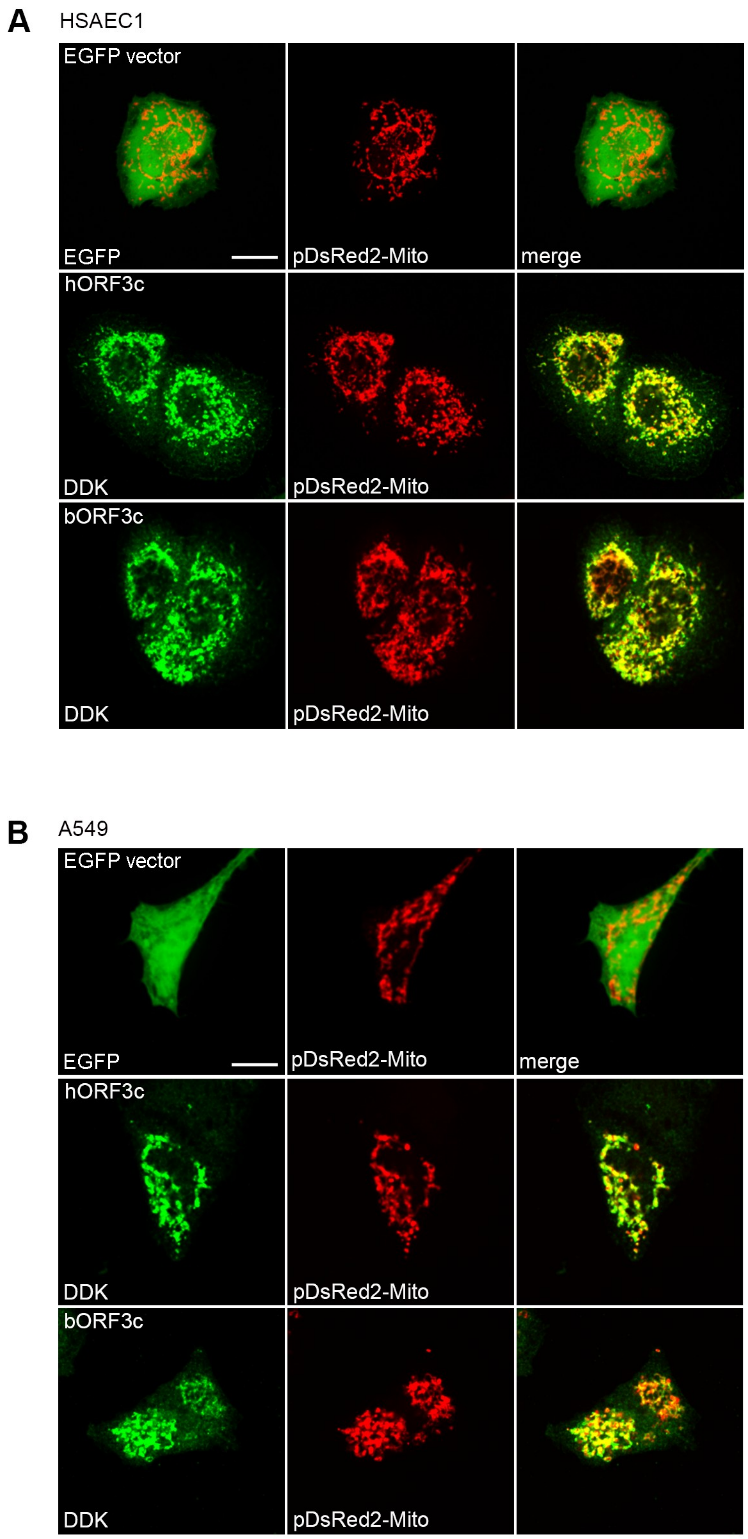

Figure S3. Mitochondrial localization of ORF3c in different cell lines, related to Figure 1.
(**A**) HSAEC1 and (**B**) A549 pulmonary cells expressing hORF3c, bORF3c or the EGFP control vector and pDsRed2-Mito to stain mitochondria were fixed and stained with the anti-DDK antibody (green), 24 h after transfection. Scale bar: 10 µm.

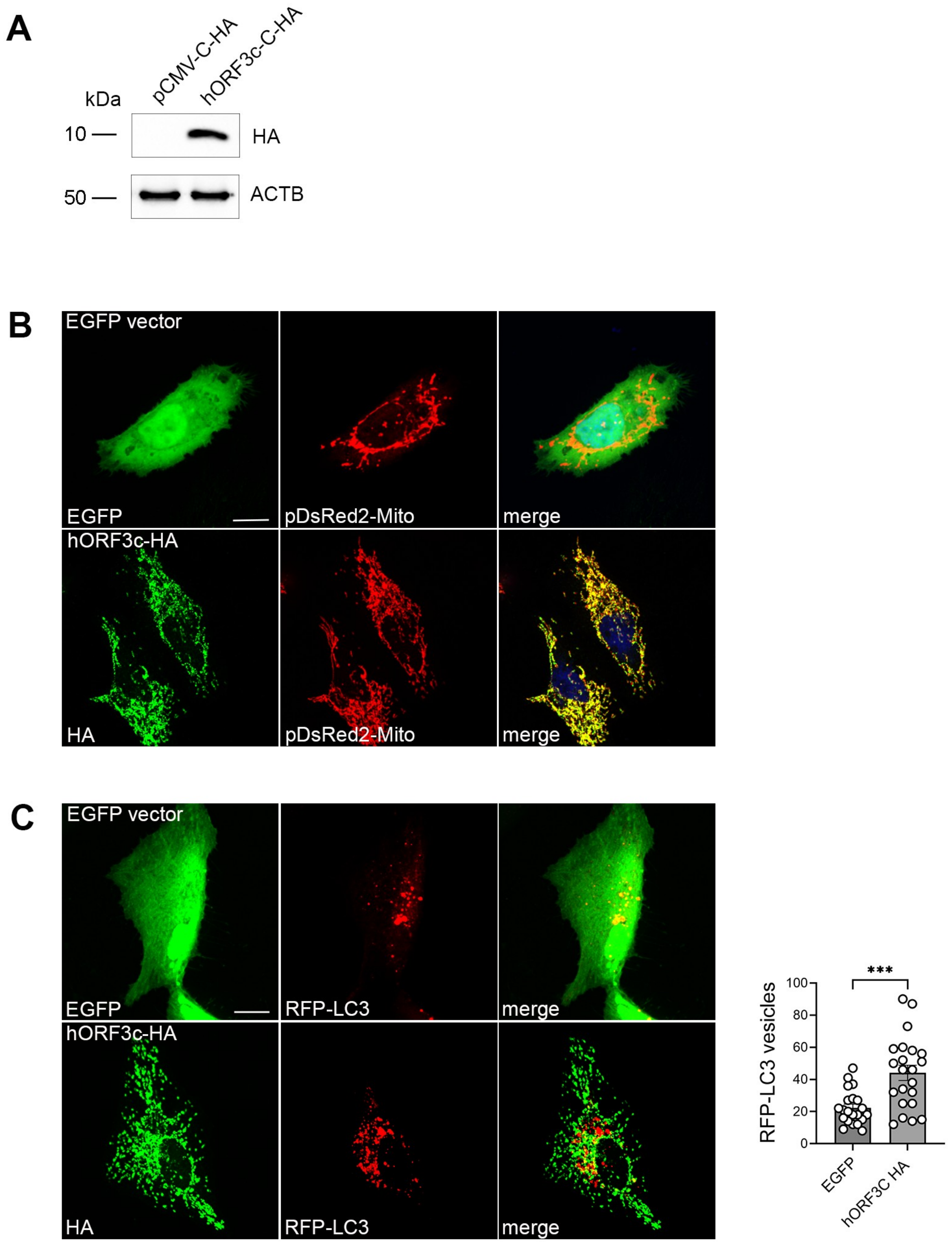

Figure S4. The tag sequence does not affect cell localization and autophagy, related to Figure 1 and Figure 4.

(**A**) HeLa cells were transfected with hORF3c-HA or with the empty vector pCMV-C-HA and total extracts were analysed by SDS-PAGE, 24 h after transfection. hORF3c was detected with anti HA antibody. (**B**) HeLa cells were transfected with hORF3c-HA or the EGFP control vector and pDsRed2-Mito to stain mitochondria, fixed and stained with the anti-HA antibody (green). Scale bar: 10 μm. (**C**) HeLa cells were transfected with hORF3c-HA or the EGFP control vector and RFP-LC3 to stain autophagosomes, fixed and stained with the anti-HA antibody (green). Scale bar: 10 μm. RFP-LC3 positive vesicles are reported in the graph (*t* test, $n>20$).

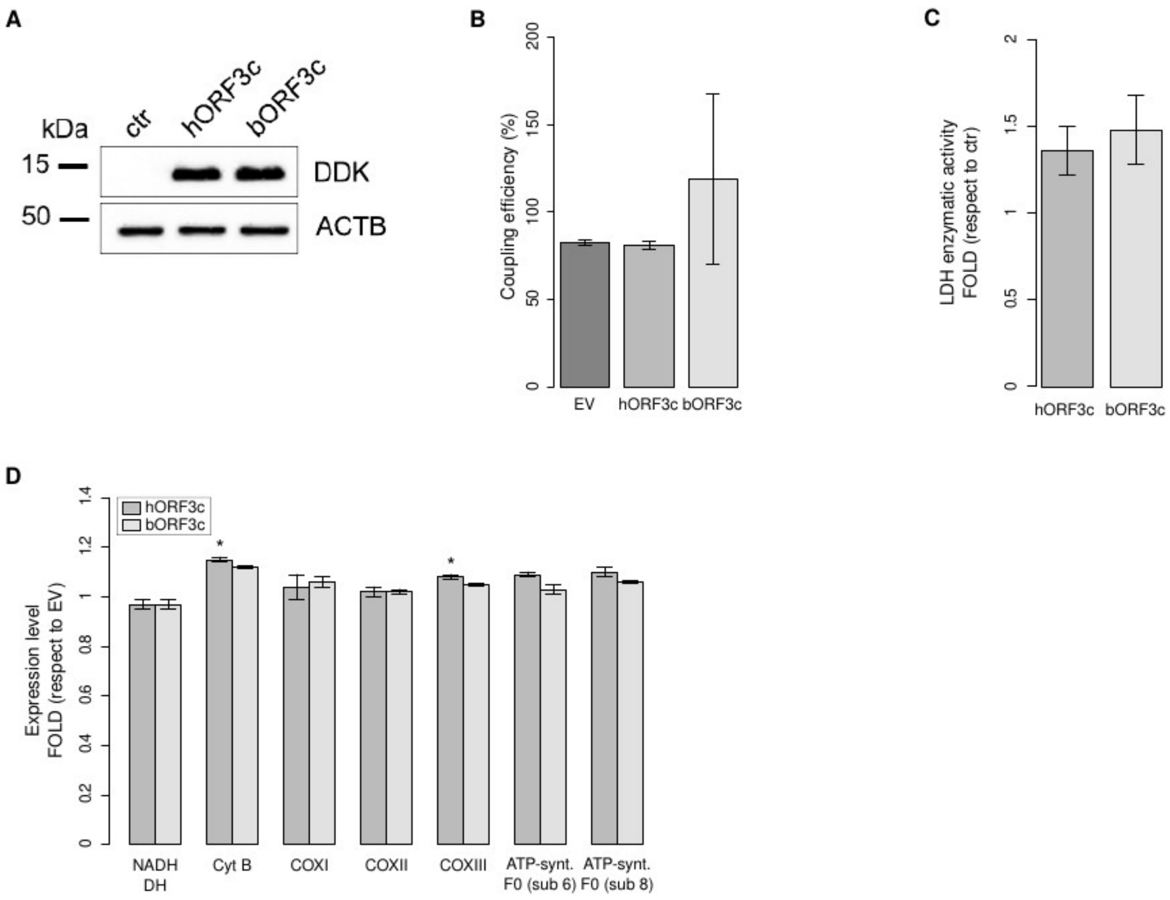

Figure S5 Additional investigations on respiratory mitochondrial metabolism, related to Figure 2.
(A) Evaluation of hORF3c and bORF3c protein expression level assayed 36 h post trasfection in HSAEC1 cell line by Western Blot analysis. Ctr refers to cells transfected with the empty vector (pCMV6-entry).
(B) Coupling efficiency in HSAEC1 cells transfected with either empty vector, hORF3c or bORF3c plasmids (36 h post trasfection). **(C)** Enzyme activity of LDH in HSAEC1 cells transfected with either hORF3c or bORF3c, compared to HSAEC1 cells transfected with an empty vector (36 h post trasfection). Results are expressed as folds with respect to control and are shown as mean ± SEM from three independent experiments (biological replicates).
(D) Quantification of basal mRNA levels by Real-Time PCR in the HSAEC1 cells transfected with hORF3c, bORF3c or with the empty vector (36 h post trasfection). The estimation of the transcript level in Real-Time PCR was carried out using the relative quantification method, normalizing the Ct values on the housekeeping beta-actin gene. Results are expressed as folds with respect to control and are shown as mean ± SEM from three independent experiments (one way ANOVA followed by Dunnett’s multiple comparison test).

A

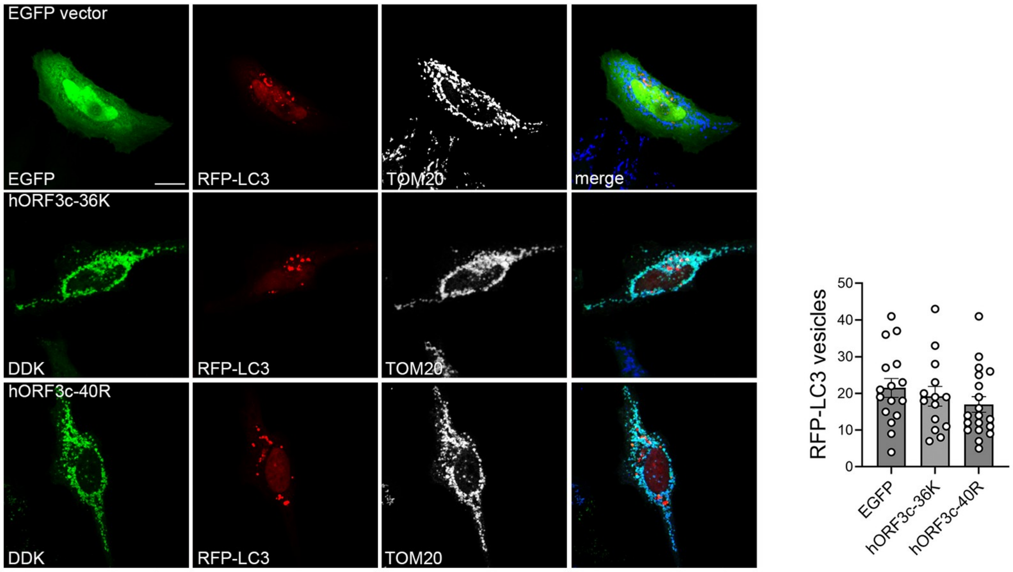

B

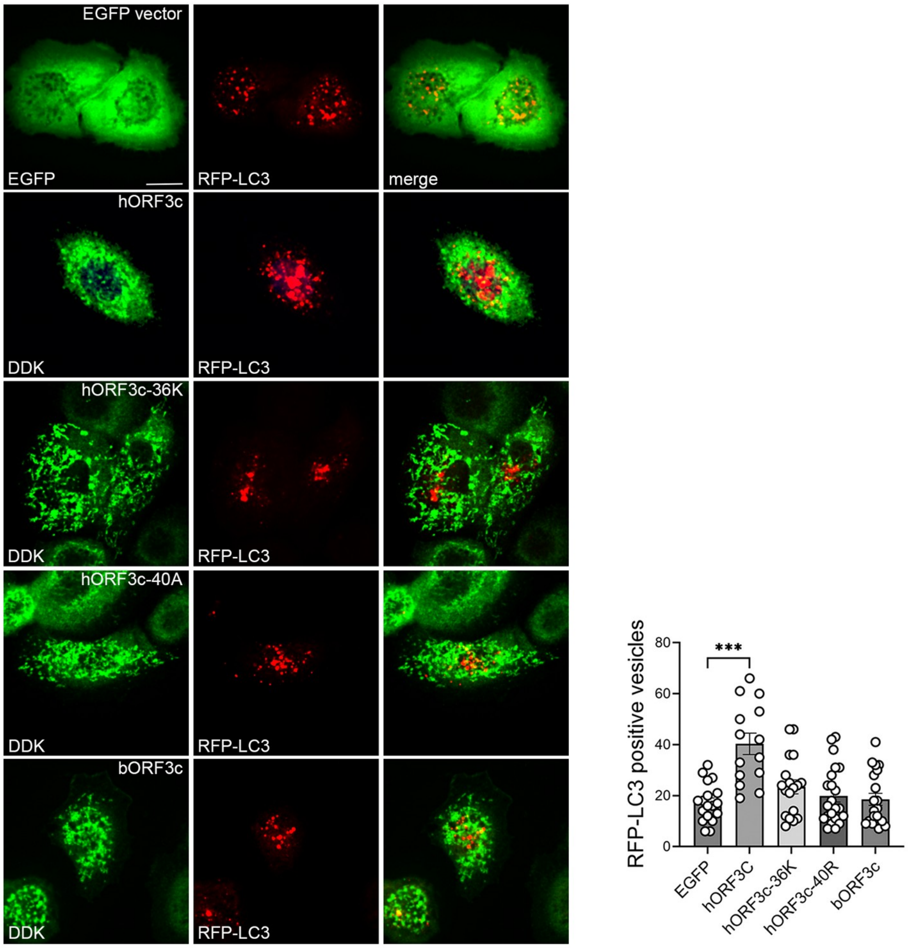

Figure S6. Mutations 36K and 40R do not affect autophagy, related to Figure 1 and Figure 4.
(**A**) HeLa cells were co-transfected with RFP-LC3B and with hORF3c-36K, hORF3c-40R or EGFP vector. Twenty-four hours post trasfection, RFP-LC3 positive vesicles were quantified and reported in the graph. (**B**) HSAEC1 cells were co-transfected with RFP-LC3B and with hORFc, hORF3c-36K, hORF3c-40R, bORF3c or EGFP vector. Twenty-four hours post trasfection, RFP-LC3 positive vesicles were quantified and reported in the graph (one way ANOVA followed by Dunnett’s multiple comparison test; *n*>15 cells).

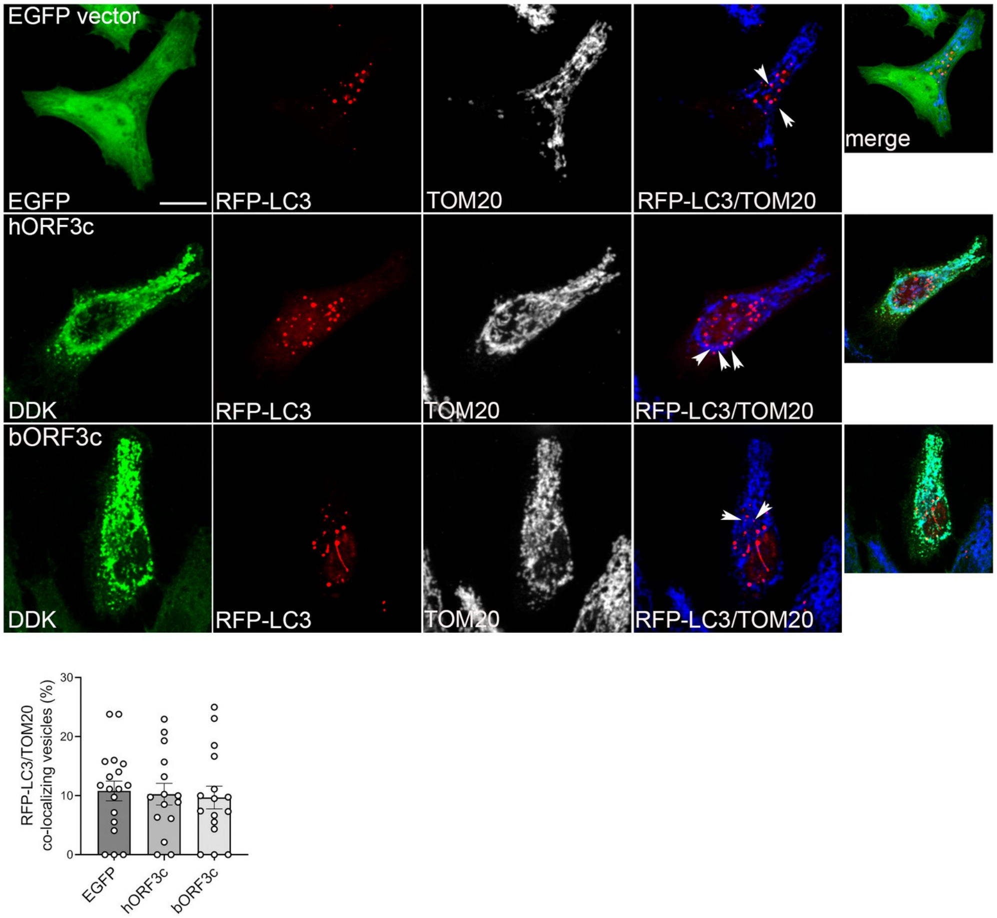

Figure S7. ORF3c expression does not induce mitophagy, related to Figure 5.
HeLa cells co-transfected with RFP-LC3B and hORF3c, bORF3c or EGFP vector were stained with anti-DDK and -TOM20 Abs. Twenty-four hours post trasfection, RFP-LC3 positive vesicles co-localizing with the mitochondrial marker TOM20 were counted, normalized on total RFP-LC3 positive vesicles and expressed as percentage (one way ANOVA followed by Dunnett's multiple comparison test; *n*=15 cells).
